# Individualized resuscitation strategy for septic shock formalized by finite mixture modeling and dynamic treatment regimen

**DOI:** 10.1186/s13054-021-03682-7

**Published:** 2021-07-12

**Authors:** Penglin Ma, Jingtao Liu, Feng Shen, Xuelian Liao, Ming Xiu, Heling Zhao, Mingyan Zhao, Jing Xie, Peng Wang, Man Huang, Tong Li, Meili Duan, Kejian Qian, Yue Peng, Feihu Zhou, Xin Xin, Xianyao Wan, ZongYu Wang, Shusheng Li, Jianwei Han, Zhenliang Li, Guolei Ding, Qun Deng, Jicheng Zhang, Yue Zhu, Wenjing Ma, Jingwen Wang, Yan Kang, Zhongheng Zhang

**Affiliations:** 1 Department of Critical Care Medicine, Guiqian International General Hospital, Guiyang, People’s Republic of China; 2 Department of Critical Care Medicine, The 8th Medical Center of Chinese, PLA General Hospital, Beijing, 100091 People’s Republic of China; 3 Department of Intensive Care Unit, Guizhou Medical University Affiliated Hospital, Guiyang, People’s Republic of China; 4 Department of Critical Care Medicine, West China Hospital of Sichuan University, Chengdu, People’s Republic of China; 5 Department of Intensive Care Unit, The First Hospital of Jilin University, Changchun, People’s Republic of China; 6 Department of Critical Care Medicine, Hebei General Hospital, Shijiazhuang, People’s Republic of China; 7 Department of Critical Care Medicine, The First Affiliated Hospital of Harbin Medical University, Harbin, People’s Republic of China; 8 General Intensive Care Unit Department, The First Affiliated Hospital of Fujian Medical University, Fuzhou, People’s Republic of China; 9 Department of Critical Care Medicine, Fu Xing Hospital, Capital Medical University, Beijing, People’s Republic of China; 10 General Intensive Care Unit, Second Affiliated Hospital of Zhejiang University, Hangzhou, People’s Republic of China; 11 Department of Critical Care Medicine, Beijing Tongren Hospital, Capital Medical University, Beijing, People’s Republic of China; 12 Department of Critical Care Medicine, Beijing Friendship Hospital, Capital Medical University, Beijing, People’s Republic of China; 13 Department of Critical Care Medicine, The First Affiliated Hospital of Nanchang University, Nanchang, People’s Republic of China; 14 Department of Critical Care Medicine, The Third Xiangya Hospital, Central South University, Changsha, People’s Republic of China; 15 Department of Critical Care Medicine, Chinese PLA General Hospital, Beijing, People’s Republic of China; 16 Surgical Intensive Care Unit, Beijing Chao-Yang Hospital, Capital Medical University, Beijing, People’s Republic of China; 17 The First Affiliated Hospital of Dalian Medical University, Dalian, People’s Republic of China; 18 Department of Intensive Care, Peking University Third Hospital, Beijing, People’s Republic of China; 19 Department of Emergency, Tongji Hospital, Tongji Medical College, Huazhong University of Science and Technology, Wuhan, People’s Republic of China; 20 Department of Critical Care Medicine, The 8th medical Center of Chinese, PLA General Hospital, Beijing, People’s Republic of China; 21 Department of Critical Care, Beijing PingGu Hospital, Capital Medical University, Beijing, People’s Republic of China; 22 Intensive Care Unit, The Hospital of Shunyi District, Beijing, People’s Republic of China; 23 Department of Critical Care Medicine, The 4th Medical Center of Chinese, PLA General Hospital, Beijing, People’s Republic of China; 24 Department of Critical Care Medicine, Shandong Provincial Hospital, Affiliated to Shandong First Medical University, Jinan, People’s Republic of China; 25 Department of Critical Care, Beijing Luhe Hospital, Capital Medical University, Beijing, People’s Republic of China; 26 Department of Critical Care, Beijing Miyun Hospital, Beijing, People’s Republic of China; 27 Intensive Care Unit, Beijing Changping District Hospital, Beijing, People’s Republic of China; 28 Department of Critical Care Medicine, West China Hospital of Sichuan University, Chengdu, People’s Republic of China; 29 Department of Emergency Medicine, Sir Run Run Shaw Hospital, Zhejiang University School of Medicine, Hangzhou, 310016 People’s Republic of China

**Keywords:** Sepsis, Dynamic treatment regime, Mortality, Fluid resuscitation

## Abstract

**Background:**

Septic shock comprises a heterogeneous population, and individualized resuscitation strategy is of vital importance. The study aimed to identify subclasses of septic shock with non-supervised learning algorithms, so as to tailor resuscitation strategy for each class.

**Methods:**

Patients with septic shock in 25 tertiary care teaching hospitals in China from January 2016 to December 2017 were enrolled in the study. Clinical and laboratory variables were collected on days 0, 1, 2, 3 and 7 after ICU admission. Subclasses of septic shock were identified by both finite mixture modeling and *K*-means clustering. Individualized fluid volume and norepinephrine dose were estimated using dynamic treatment regime (DTR) model to optimize the final mortality outcome. DTR models were validated in the eICU Collaborative Research Database (eICU-CRD) dataset.

**Results:**

A total of 1437 patients with a mortality rate of 29% were included for analysis. The finite mixture modeling and *K*-means clustering robustly identified five classes of septic shock. Class 1 (baseline class) accounted for the majority of patients over all days; class 2 (critical class) had the highest severity of illness; class 3 (renal dysfunction) was characterized by renal dysfunction; class 4 (respiratory failure class) was characterized by respiratory failure; and class 5 (mild class) was characterized by the lowest mortality rate (21%). The optimal fluid infusion followed the resuscitation/de-resuscitation phases with initial large volume infusion and late restricted volume infusion. While class 1 transitioned to de-resuscitation phase on day 3, class 3 transitioned on day 1. Classes 1 and 3 might benefit from early use of norepinephrine, and class 2 can benefit from delayed use of norepinephrine while waiting for adequate fluid infusion.

**Conclusions:**

Septic shock comprises a heterogeneous population that can be robustly classified into five phenotypes. These classes can be easily identified with routine clinical variables and can help to tailor resuscitation strategy in the context of precise medicine.

**Supplementary Information:**

The online version contains supplementary material available at 10.1186/s13054-021-03682-7.

## Introduction

Septic shock is a leading cause of mortality and morbidity in the intensive care unit (ICU). The shock status should be corrected as soon as possible to prevent subsequent development of multiple organ dysfunctions [1–3]. The resuscitation of septic shock at the initial phase involves fluid infusion and use of vasoactive agents such as norepinephrine, dopamine and dobutamine [4, 5]. Although the Surviving Sepsis Campaign guidelines recommend several goals (i.e., urine output, mean blood pressure and ScvO_2_) to guide resuscitation [6, 7], the specific strategy must be individualized because the responses to a given intervention can vary greatly among septic shock patients. For example, some patients with sepsis-induced acute kidney injury may respond well to fluid challenge, while others may further develop renal failure after fluid resuscitation [8, 9]. The clinical heterogeneity must be accounted for in both clinical practice and clinical trial design. Since sepsis and/or septic shock is a heterogeneous clinical syndrome, many clinical trials targeting sepsis population usually result in neutral findings [2, 10, 11]. In these trials, some patients may benefit from a certain intervention, but others will be harmed by the intervention, resulting in a neutral effect in the overall population. Thus, numerous efforts have been made to explore the heterogeneity of sepsis.

Sepsis has been found to be consisted of several phenotypes, though specific class membership assignments are different across studies [12–18]. By using clinical trial data from 1696 patients, Gårdlund B and colleagues identified six classes of septic shock [19]. Alternatively, the use of temperature trajectory was able to identify four classes of sepsis [20]. The identified classes were investigated for their responses to different treatments. Our previous results showed that these clinical subclasses have different responses to the amount of fluid infusion [14]. By utilizing randomized clinical trial data, the proportion of RCTs reporting benefit, harm or no effect changed considerably by varying the proportion of subclasses [21]. These results indicated that the subclasses of sepsis should be considered in designing clinical trials because of their differing responses to fluid strategies. However, previous studies primarily explored subclasses of sepsis using cross-sectional data, ignoring the transition pattern of classes and the sequential treatment decisions. In real clinical practice, septic shock is managed with sequential treatment decisions. In other words, the treatment decision making in the current stage should consider not only the current status but also previous responses to the treatment. Unfortunately, such a clinical practice pattern has not been formalized with mathematical modeling.

Previous studies have explored the feasibility of utilizing high-granularity dataset to develop sequential decision rules of resuscitation for septic shock. For example, Komorowski M and colleagues developed a reinforcement learning algorithm to determine the sequential rules of treatment strategy [22]. Our study group utilized dynamic treatment regime (DTR) model to develop a sequential treatment strategy [23]. However, these models are of high granularity with limited explainability. To make the model more explainable, the present study firstly classified septic shock into several classes by using non-supervised learning algorithms. Then, the classification system was integrated in a DTR model to develop a sequential treatment rule for fluid volume and vasopressor dosing [24]. The optimal treatment strategy was compared with the actual strategy through days 0–7, and risk factors for fluid overload and norepinephrine overdosing were explored. We hypothesized that several classes of septic shock could be robustly identified and DTR model was able to identify optimal treatment strategy for these classes. The differences between actual and optimal treatment strategy varied across different classes.

## Methods

### Study design and setting

The study was conducted in 25 tertiary care teaching hospitals in China from January 2016 to December 2017. Participants were retrospectively enrolled by reviewing the electronic medical records, and the patients were followed up during the hospital stay. A site investigator was responsible for this study in each ICU. Additionally, a clinical research coordinator (CRC) was assigned to each hospital to ensure the quality of data collection. The study was approved by the ethics committee of the 8th Medical Center of General Hospital of Chinese People’s Liberation Army (approval no. 309201906171117). The written informed consent was waived by the ethics committee because the study did not involve any interventions. Data were deidentified and stored in an encrypted computer. This study was registered on the Chinese Clinical Trial Registry (Registration No. ChiCTR1900024418).

### Participants

Sepsis was defined as suspected or documented infection plus an increase in the Sequential [Sepsis-related] Organ Failure Assessment (SOFA) score of 2 points or more. Baseline SOFA score was extracted from past medical history. SOFA score was assumed to be 0 if a subject had no known comorbidities or baseline laboratory measurements. Patients with septic shock were then identified by a vasopressor requirement to maintain a mean arterial pressure of 65 mmHg or greater and serum lactate level greater than 2 mmol/L (> 18 mg/dL) in the absence of hypovolemia (e.g., as determined by the CRCs from participating center based on clinical findings such as increased HR, low CVP, decreased blood pressure and pale, cool and clammy skin) [25]. Patients were enrolled if they had septic shock on ICU admission. Exclusion criteria included pregnancy, age younger than 18 years old, terminal illness or malignancy with do-not-resuscitate order, concomitant acute myocardial infarction or pulmonary embolism with hemodynamic compromise, conditions of immunodeficiency (i.e., hematological malignancy, neutropenia, organ transplantation) and surgical source of infection not controlled.

### Variables

Clinical and laboratory variables were collected on days 0, 1, 2, 3 and 7 after ICU admission. For numeric variables, the minimum and maximum values during each of these days were collected. Demographic and baseline clinical data included age, gender, type of patient (elective surgery, emergency surgery, non-surgical), weight on admission, comorbidity and site of infection (abdominal, thorax, brain, blood stream, soft tissue and urinary tract infection). Vital signs of body temperature, arterial blood pressure, heart rate and respiratory rate were recorded. Acute physiology and chronic health evaluation (APACHE) II score was calculated within 24 h after ICU admission. Laboratory variables included pH, HCO_3_, serum lactate, hemoglobin (HB), hematocrit (HCT), PaCO_2_, P/F ratio, base excess (BE), platelet count, red blood cell distribution width (RDWCV), serum creatinine and total bilirubin. Fluid intake volume was calculated by summing up all crystalloids, colloids, blood products, nasogastric (NG) water, NG feed, parenteral nutrition and fluid intake associated with IV drugs administration. Fluid intake and output were measured on the daily basis. Vasopressors including norepinephrine, epinephrine, dopamine and dobutamine were recorded. Vasopressors were converted to equivalent dose of norepinephrine by the equation (all in mcg/kg/min, except vasopressin in units/min) [26]:

$$\begin{aligned} {\text{Norepinephrin}}\,{\text{eequivalent}} & = {\text{ Norepinephrine }} + {\text{ Epinephrine }} \\ & \quad + {\text{ Phenylephrine/1}}0 \, + {\text{ Dopamine/1}}00 \, \\ & \quad + {\text{ Metaraminol/8 }} + {\text{ Vasopressin}}\,\times {2}.{5 } + {\text{ Angiotensin}}\,{\text{ II}}\times 10. \\ \end{aligned}$$

Patients with 20% or more of missing values were excluded from analysis. For other patients with missing values, the longitudinal data were imputed by firstly applying last observation carried forward (LOCF) and then next observation carried backward (NOCB) methods [27].

### Classes of septic shock

**Fig. 1:**
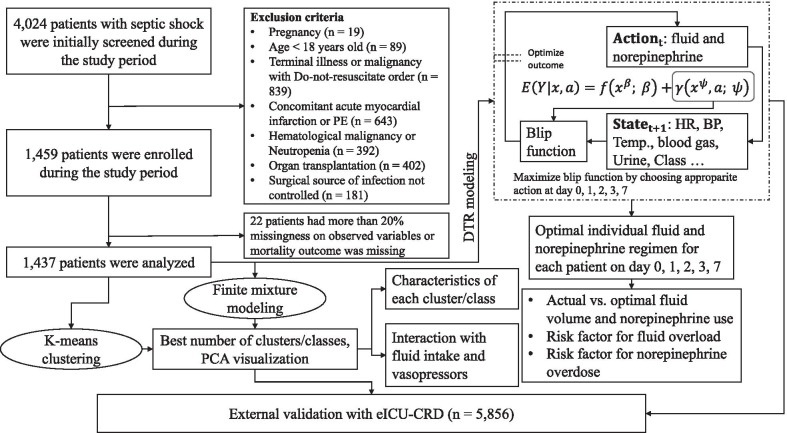
Flowchart of patient enrollment and schematic illustration of analysis workflow. A total of 1437 septic shock patients were analyzed. The first step is to identify classes of septic shock by both finite mixture modeling and *K*-means clustering. Clinical characteristics of each class were compared. Interaction between class membership and fluid volume or epinephrine dose was explored in a multivariable Cox model with time-varying covariates. A significant effect of interaction means different therapeutic effects across classes. Another thread of our analysis is to estimate optimal fluid volume and norepinephrine dose using dynamic treatment regimen. The key of the modeling is to construct a blip function that can help to tailor optimal dosing strategy based on current patient status and historical response to the intervention. It returns a sequential decision policy to optimize the final outcome. The optimal fluid volume or epinephrine dose was then compared with the actual strategy, and relevant risk factors can be explored for fluid overload or norepinephrine overdosing

The classes of septic shock were explored by using finite mixture modeling (FMM) by assuming Gaussian distributions of feature variables, and variances were constrained to be equal across classes, and covariances were fixed to 0 (Fig. 1). Correlation between feature variables was examined using Pearson’s correlation analysis. We removed highly correlated variables by domain knowledge. Candidate variables representing several key pathophysiological domains were included for FMM, such as baseline demographics (age, weight), disease severity (APACHE II), vital signs (SBP, DBP, HR, temperature, RR), tissue perfusion (lactate), internal environment (BE, pH, HCO_3_), respiration (PaCO_2_, PaO_2_, PF), inflammatory responses (CRP, RDWCV), hematology (platelet) and renal function (urine output, creatinine). PaO_2_, CRP and DBP were removed due to their correlation with other variables with correlation coefficient > 0.7. The FMM was fit to the combined dataset of feature vectors from all patients across days 0, 1, 2, 3 and 7, while allowing class transition across ICU days. The best number of classes was determined by both statistics and clinical importance. Lower values of AIC and SABIC and higher values of entropy were considered as better model fit. Bootstrap likelihood ratio test was performed to compare whether *k*-class model was better than (*k *− 1)-class model [28]. The minimum number of patients should be over 4% of the entire study population. The minimum probability of assigning to one class should be over 0.8; otherwise, the class membership is considered as unstable. The best number of classes was also confirmed by the *k*-means clustering analysis. Statistics such as cubic clustering criterion (CCC), Calinski and Harabasz (CH) index, Davies and Bouldin (DB) index, Hartigan, Krzanowski and Lai (KL) index, Marriot, Rubin and TraceW were reported because they were easily implemented in the package NbClust (V3.0) [29]. PCA was also used to visualize the identified classes to show that the classes of septic shock can be visualized in lower-dimensional space.

Characteristics of each class were compared using Kruskal–Wallis rank sum test or analysis of variance (ANOVA) for numeric data, and Chi-square test or Fisher’s exact test for categorical data [30]. Interactions between class membership and fluid volume ($\mathrm{Class}\times \mathrm{Fluid}\,\mathrm{Volume}$) or norepinephrine use ($\mathrm{Class}\times \mathrm{Norepinephrine}\, \mathrm{Dose}$) were explored in two multivariable Cox regression models with time-varying covariates. Other covariates including comorbidity, APACHE II, urine output, creatinine and age were adjusted for. A statistically significant interaction might indicate potential differing effect size of the intervention (either fluid volume or norepinephrine equivalence dose) on survival outcome in different classes.

### Dynamic treatment regimen modeling

Optimal dynamic treatment regimes can be inferred from observational medical data using reinforcement learning algorithm [31, 32]. Alternatively, DTR can also be used to estimate optimal treatment strategy across multiple treatment stages, so that the final clinical outcome can be optimized [33, 34]. In this study, the treatments are continuous variables including fluid volume intake and norepinephrine dosing. The regression-based method was used for estimating the optimal dosing strategy across days 0, 1, 2, 3 and 7 after ICU admission. More specifically, the mortality outcome $E\left(Y|x,a\right)$ was modeled in terms of treatment-free model $f\left({x}^{\beta }; \beta \right)$ and a blip function $\gamma \left({x}^{\psi }, a; \psi \right)$: $E\left(Y|x,a\right)=f\left({x}^{\beta }; \beta \right)+\gamma \left({x}^{\psi }, a; \psi \right)$, where $x$ is a vector of covariates and $a$ is the treatment strategy. ${x}^{\beta }$ and ${x}^{\psi }$ are subsets of observed covariates vector $\mathbf{x}$. The blip function is parameterized in terms of $\psi$ and characterizes the treatment effect. DTR model also requires specification of a treatment model, which is a propensity score for receiving treatment: $\pi \left(a|x\right)={f}_{A|x}\left(a|x\right)$. The goal of parameter estimation is to optimize the final outcome $Y$ in a sequential manner. The estimation was performed by dynamic weighted ordinary least squares [33, 34]. The results of the DTR model would return individualized optimal dosing strategy for both fluid volume and norepinephrine dosing across days 0, 1, 2, 3 and 7. Then, the actual treatment strategy was compared to the optimal treatment strategy. Fluid overloading was defined as those receiving > 1000 mL/day than the optimal volume, and norepinephrine overdosing was those receiving > 0.1 mcg/kg/min than the optimal dose. Risk factors for fluid and norepinephrine overdosing were explored by using stepwise backward elimination and forward selection logistic regression models with AICs. (More details are given in Additional file 1.) All analyses were performed using R (version 4.0.1), and codes are available in Additional file 2.

### External validation in the eICU-CRD dataset

The eICU Collaborative Research Database (eICU-CRD), which was a multi-center intensive care unit (ICU) database with high-granularity data for over 200,000 admissions to ICUs monitored by eICU Programs across the USA, was utilized for model validation [35]. Septic shock patients were defined as those with the admission diagnosis of sepsis plus the use of vasopressor including norepinephrine, dopamine and epinephrine. The same feature variables were used for FMM and DTR modeling. The classification of septic shock was validated in the eICU-CRD database. The cluster membership for new samples in eICU-CRD was determined by the highest posterior probability of the class membership. The FMM can also be helpful for the application our model to external dataset. Downstream DTR validation was performed by predicting the optimal treatment strategy for patients in the eICU-CRD. Then, the optimal strategy was validated by the fact that it was associated with the lowest mortality.

## Results

### Participants

A total of 4024 patients with septic shock were screened during the study period. After application of exclusion criteria, 1459 patients were enrolled for the study. However, 22 patients were further excluded because of predefined proportion of missing values. As a result, we included 1437 patients for subsequent analysis (Fig. 1). The median age of the study population was 67 years (IQR 54 to 78 years). There were more male (912/1437, 63%) than female patients. The median APACHE II was 22 (IQR 16–27). The primary site of infection included thorax (48%), abdomen (38%), UTI (7%) and soft tissue (3%). Medical patients accounted for 56% of all patients, followed by emergency surgery (32%) and elective surgery patients (12%).

### Classes of septic shock

**Fig. 2:**
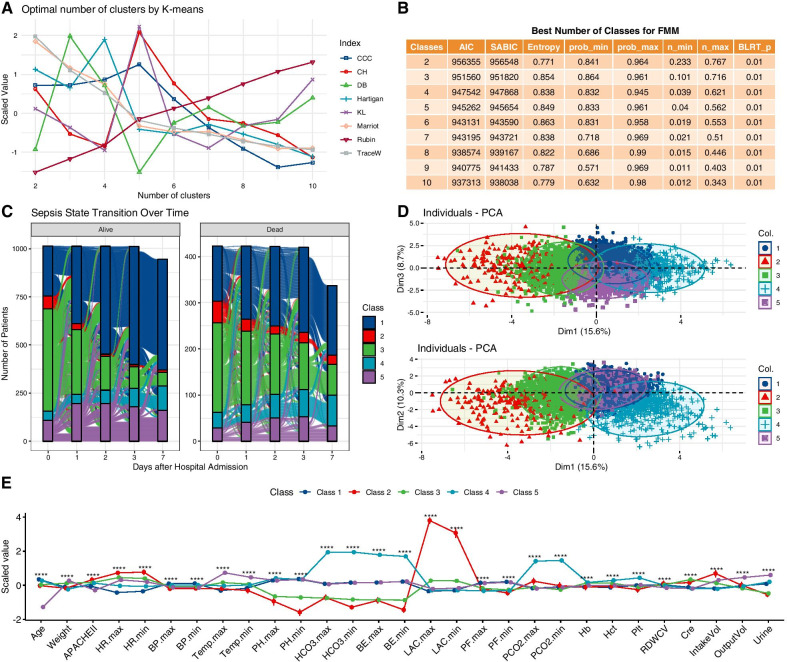
Classes of septic shock. **A** Optimal number of clusters by *K*-means clustering. The statistics were scaled for better visualization. Statistics such as CCC, CH and KL showed the highest value for five clusters; lower value of DB indicates better fit which also supports the existence of five clusters. Some statistics utilize elbow point to identify the best number of clusters such as Hartigan, Marriot and TraceW. These statistics consistently indicated an elbow joint at 5-class. **B** Metrics for choosing the best number of classes for FMM. **C** Class membership transition over days 0, 1, 2, 3 and 7. It is noted that most patients in class 2 (critical class) on day 0 would transition to other classes, indicating improvement in these critical cases. Patients who transitioned to class 2 were more likely to die. **D** PCA showing that the five classes can be visually separated by the first three principal components. One point represents one sample (one data point per patient day). **E** Characteristics of the five classes identified by FMM. All numeric values were scaled (i.e., centered on mean and divided by standard deviation) for better visualization on the vertical axis. Seventeen variables were used for FMM training, but 29 variables are displayed to give a comprehensive clinical characteristics for these classes. Class 1 is the largest class over all study days with all variables in average value (the baseline class). Class 2 is characterized by poor tissue perfusion and multiple organ failure and can be called the critical class. Class 3 is characterized by highest serum creatinine and metabolic acidosis and can be called renal dysfunction class. Class 4 is characterized by the highest PaCO2 (60; IQR 50–77 mmHg) and low PF ratio (169; IQR 118–232 mmHg) and can be designated as respiratory failure class. Class 5 is characterized by young age, low mortality and well-preserved renal function and can be considered as the mild class. *FMM* finite mixture modeling, *CCC* cubic clustering criterion, *CH* Calinski and Harabasz index, *DB* Davies and Bouldin index, *KL* Krzanowski and Lai index. ****< 0.001

A total of 17 features (age, weight, HR, APACHE II, SBP, temperature, pH, HCO_3_, LAC, BE, PF, PaCO2, HCT, platelet, RDWCV, creatinine, urine) were included for FMM. The values of AIC and SABIC declined form 2-class to 8-class models, but the smallest class contained less than 3% patients from 6-class to 8-class models (Fig. 2B). Thus, the 5-class model was considered as the best model. Furthermore, the 5-class model showed an entropy of 0.849, which was among the largest values. The 5-class model was confirmed by *k*-means clustering analysis (Fig. 2A). The class membership transition is shown in Fig. 2C, showing that patients can move from class to class over ICU days. Patients transitioned to class 1 were more likely to survive on hospital discharge. The five classes could be well separated in the first three principal components (explaining 15.6%, 10.3% and 8.7% of the total variance, Fig. 2D). Characteristics of the five classes are visualized in Fig. 2E, and statistical comparisons are shown in Table 1. Class 1 is the largest class over all study days (it is not the largest class on day 0 as shown in Table 1) with all clinical features around the population mean (the baseline class). Class 2 is characterized by poor tissue perfusion (high serum lactate level: 11.10; IQR 9.05–14.25 mmol/L) and the highest mortality rate (41%) and can be called the critical class. Class 3 is characterized by highest serum creatinine and metabolic acidosis and can be called renal dysfunction class (Fig. 2E). Class 4 is characterized by the highest PaCO_2_ (60; IQR 50–77 mmHg) and low PF ratio (169; IQR 118–232 mmHg) and can be designated as respiratory failure class. Class 5 is characterized by young age (42; IQR 30–51 years) and low mortality (21%) and can be considered as the mild class.

**Table 1:** Comparisons of clinical and laboratory variables across classes on day 0

Variables	Total (*n* = 1437)	1 (*n* = 380)	2 (*n* = 115)	3 (*n* = 723)	4 (*n* = 81)	5 (*n* = 138)	*p*
Age (years), median (IQR)	67.00 (54.00, 78.00)	72.50 (62.00, 82.00)	65.00 (55.00, 75.50)	67.00 (55.50, 77.00)	77.00 (61.00, 84.00)	**42.00 (30.00, 51.00)**	< 0.001
Gender, male (%)	912 (63)	245 (64)	71 (62)	450 (62)	59 (73)	87 (63)	0.425
Weight (kg), median (IQR)	64.00 (56.00, 70.00)	64.00 (54.00, 69.00)	64.00 (58.00, 70.00)	64.00 (60.00, 71.00)	62.00 (53.00, 65.00)	65.00 (60.00, 70.00)	< 0.001
*Comorbidity, n (%)*							< 0.001
CRF	51 ( 4)	13 (3)	2 (2)	27 (4)	5 (6)	4 (3)	
None	681 (47)	162 (43)	56 (49)	333 (46)	35 (43)	95 (69)	
Diabetes	288 (20)	76 (20)	28 (24)	145 (20)	18 (22)	21 (15)	
Hypertension	336 (23)	99 (26)	26 (23)	176 (24)	20 (25)	15 (11)	
CAD	81 ( 6)	30 (8)	3 (3)	42 (6)	3 (4)	3 (2)	
*Site of infection, n (%)*							< 0.001
Abdomen	544 (38)	112 (29)	46 (40)	332 (46)	7 (9)	47 (34)	
Thorax	690 (48)	226 (59)	49 (43)	281 (39)	70 (86)	64 (46)	
Brain	13 ( 1)	6 (2)	1 (1)	1 (0)	1 (1)	4 (3)	
Blood	36 ( 3)	8 (2)	3 (3)	17 (2)	3 (4)	5 (4)	
Soft tissue	50 ( 3)	7 (2)	6 (5)	26 (4)	0 (0)	11 (8)	
UTI	104 ( 7)	21 (6)	10 (9)	66 (9)	0 (0)	7 (5)	
*Type, n (%)*							< 0.001
Medical	800 (56)	232 (61)	67 (58)	361 (50)	69 (85)	71 (51)	
Elective surgery	179 (12)	52 (14)	15 (13)	89 (12)	3 (4)	20 (14)	
Emergent surgery	458 (32)	96 (25)	33 (29)	273 (38)	9 (11)	47 (34)	
Hours on day 0, median (IQR)	13.00 (9.00, 17.00)	13.50 (10.00, 17.00)	12.50 (8.00, 19.00)	13.00 (9.00, 17.00)	13.40 (8.00, 18.00)	14.40 (10.62, 17.88)	0.119
MV, n (%)	1227 (85)	329 (87)	104 (90)	600 (83)	78 (96)	116 (84)	0.007
APACHEII, median (IQR)	22.00 (16.00, 27.00)	21.00 (14.00, 26.00)	25.00 (19.00, 30.00)	22.00 (17.00, 28.00)	23.00 (17.00, 26.00)	20.00 (14.00, 24.75)	< 0.001
Use of norepinephrine, *n* (%)	1259 (88)	312 (82)	108 (94)	659 (91)	65 (80)	115 (83)	< 0.001
Maximum HR (/min), Mean ± SD	122.25 ± 23.46	108.70 ± 21.08	133.97 ± 19.78	126.72 ± 22.63	119.22 ± 22.20	128.18 ± 21.39	< 0.001
Maximum SBP (mmHg), median (IQR)	132.00 (113.00, 147.00)	137.00 (120.00, 150.00)	128.00 (110.00, 147.50)	130.00 (110.00, 145.00)	136.00 (120.00, 150.00)	130.50 (111.00, 142.75)	0.001
Minimum SBP (mmHg), median (IQR)	87.00 (74.00, 98.00)	91.00 (77.00, 102.00)	**83.00 (70.00, 98.00)**	86.00 (71.00, 98.00)	88.00 (74.00, 98.00)	89.00 (78.00, 98.20)	0.002
Temperature (℃), median (IQR)	37.50 (36.90, 38.40)	37.30 (36.80, 37.80)	37.40 (36.70, 38.50)	37.60 (37.00, 38.50)	37.50 (37.00, 38.20)	38.50 (37.60, 39.20)	< 0.001
Minimum pH, median (IQR)	7.35 (7.28, 7.41)	7.40 (7.37, 7.45)	7.24 (7.14, 7.31)	7.32 (7.26, 7.37)	7.38 (7.30, 7.47)	7.41 (7.37, 7.45)	< 0.001
Minimum HCO3, median (IQR)	20.60 (17.00, 23.40)	23.20 (21.90, 25.70)	15.30 (11.40, 18.45)	18.00 (15.60, 20.50)	34.30 (32.60, 37.50)	23.10 (21.63, 25.20)	< 0.001
Minimum BE, median (IQR)	− 4.20 (− 7.90, − 0.60)	− 0.70 (− 2.40, 1.60)	− 11.00 (− 14.90, − 6.75)	− 6.90 (− 9.50, − 4.44)	8.90 (5.00, 12.60)	− 0.80 (− 2.68, 1.00)	< 0.001
Maximum lactate (mmol/L), median (IQR)	2.70 (1.80, 4.60)	1.90 (1.40, 2.50)	**11.10 (9.05, 14.25)**	3.40 (2.10, 5.00)	1.90 (1.40, 2.90)	2.00 (1.63, 2.98)	< 0.001
Minimum PF, median (IQR)	199.00 (135.17, 258.00)	210.00 (160.00, 280.00)	174.00 (99.50, 219.91)	187.50 (130.00, 250.00)	**169.00 (117.83, 232.00)**	210.00 (153.32, 297.79)	< 0.001
Maximum PaCO_2_ (mmHg), median (IQR)	39.00 (33.00, 46.00)	40.00 (35.00, 44.05)	41.00 (31.50, 47.60)	36.20 (30.25, 44.00)	**60.00 (50.00, 77.00)**	38.00 (33.00, 42.80)	< 0.001
Hb (g/l), median (IQR)	103.00 (85.00, 124.00)	97.00 (81.00, 115.00)	100.00 (85.50, 125.00)	107.00 (88.00, 126.00)	107.00 (89.00, 127.00)	105.50 (87.25, 129.50)	< 0.001
Hct (%), median (IQR)	29.90 (26.30, 36.10)	27.90 (26.00, 32.90)	30.00 (26.30, 36.65)	31.40 (26.30, 37.25)	32.00 (26.30, 38.90)	29.00 (26.30, 36.50)	< 0.001
Platelet ($\times {10}^{9}/\mathrm{L}$), median (IQR)	136.00 (81.00, 212.00)	148.00 (89.75, 214.25)	107.00 (47.00, 182.00)	131.00 (75.00, 200.00)	188.00 (122.00, 267.00)	146.50 (90.50, 236.75)	< 0.001
RDWCV, median (IQR)	14.00 (13.00, 16.00)	14.00 (13.00, 16.00)	14.00 (13.00, 15.40)	14.00 (13.00, 15.00)	14.00 (13.00, 15.00)	14.00 (13.00, 15.00)	0.108
Serum creatinine (mmol/L), median (IQR)	113.60 (71.00, 203.00)	92.05 (61.86, 134.85)	175.80 (105.65, 265.48)	**138.00 (87.74, 236.15)**	76.00 (51.80, 121.00)	79.55 (56.08, 115.84)	< 0.001
Intake volume (mL), median (IQR)	2866.00 (1900.00, 3976.00)	2480.00 (1712.00, 3457.25)	3667.00 (2102.50, 5521.00)	2962.00 (1926.00, 4045.00)	2571.00 (1806.15, 3200.50)	3448.50 (2131.25, 5115.50)	< 0.001
Output volume (mL), median (IQR)	1440.00 (800.00, 2415.00)	1450.50 (863.00, 2219.25)	1360.00 (480.00, 2414.50)	1320.00 (745.00, 2280.00)	1314.00 (628.00, 1930.00)	2537.00 (1410.75, 3545.00)	< 0.001
Urine output (mL), median (IQR)	970.00 (400.00, 1695.00)	1150.00 (646.25, 1801.25)	470.00 (206.00, 1420.00)	795.00 (320.00, 1422.50)	950.00 (515.00, 1740.00)	1835.00 (1092.50, 2990.00)	< 0.001
Hospital LOS, median (IQR)	19.00 (11.00, 32.00)	20.50 (12.00, 34.00)	19.00 (8.00, 36.50)	18.00 (10.00, 30.00)	22.00 (14.00, 36.00)	20.00 (12.00, 34.75)	0.019
ICU LOS, median (IQR)	9.00 (5.00, 17.00)	10.00 (6.00, 21.00)	9.00 (5.00, 16.00)	8.00 (5.00, 15.00)	14.00 (7.00, 23.00)	10.00 (5.25, 18.75)	< 0.001
Mortality, *n* (%)	423 (29)	120 (32)	**47 (41)**	193 (27)	34 (42)	**29 (21)**	< 0.001

Bold significances are shown in the last column (*p* < 0.001)

All variables were recorded on day 0. *LOS* length of stay, *IQR* interquartile range, *CRF* chronic renal failure, *CAD* coronary artery diseases, *UTI* urinary tract infection, *HR* heart rate, *SD* standard deviation, *ICU* intensive care unit, *RDWCV* red blood cell distribution width, *BE* base excess, *Hct* hematocrit

### Differing therapeutic effect in classes of septic shock

**Fig. 3:**
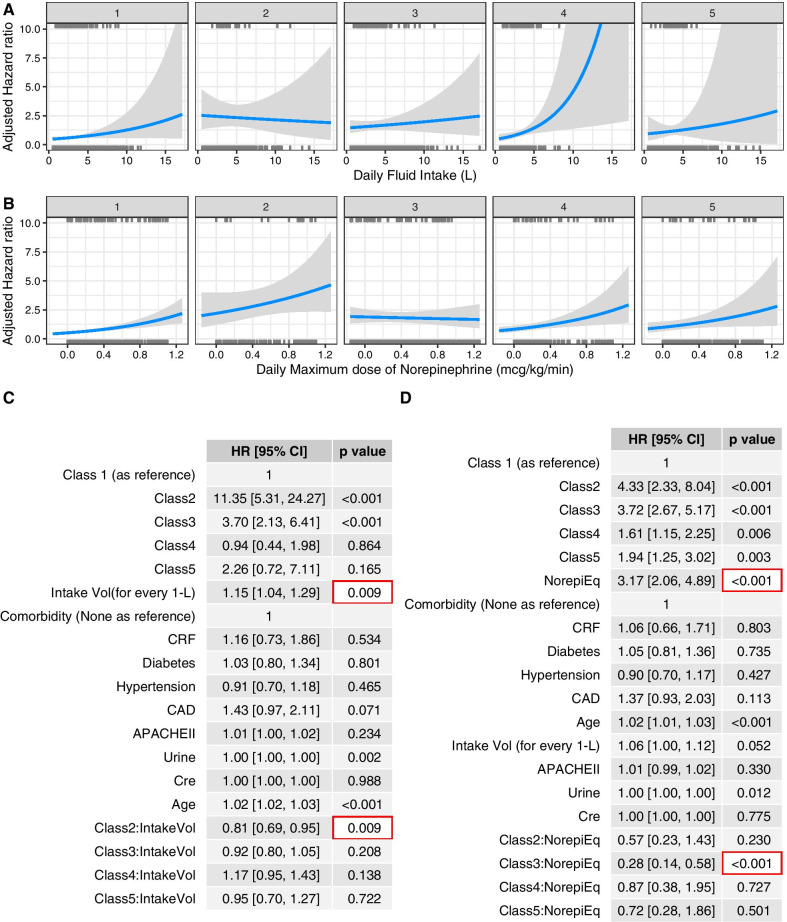
Multivariable Cox regression model with time-varying covariates. Relative hazard could be varying over time and our model reported the average value for a given dose. **A** Fluid volume and risk of mortality stratified by class membership. More fluid administration was associated with reduced risk of mortality in class 2 (Critical class). **B** Daily maximum dose of norepinephrine and hazard ratio. While more norepinephrine was associated with increased risk of mortality in overall population, greater dose of norepinephrine was associated with reduced risk of mortality in class 3 (respiratory failure class). The gray area indicates the 95% confidence interval, and the small bars on horizontal axis indicate sample points. **C** Multivariable regression model showed significant interaction between class membership and fluid volume. There was significant interaction between fluid intake and class 2 (critical class) membership (HR 0.81; 95% CI 0.69–0.95). **D** Larger dose of norepinephrine was associated with increased instantaneous hazard in the main effect (HR 3.17; 95% CI 2.06–4.89). There was significant interaction between class 3 and norepinephrine dose (HR for interaction: 0.28; 95% CI 0.14–0.58; *p* < 0.001). HRs for comorbidities were reported with the None comorbidity as reference. *HR* hazard ratio, *CI* confidence interval, *CRF* chronic renal failure, *CAD* coronary artery diseases, *NorepiEq* norepinephrine equivalence dose in mcg/kg/min, *Cre* creatinine in mg/dl, *APACHEII* Acute Physiology and Chronic Health Evaluation II, *Intake Vol* daily intake volume in liters

In multivariable Cox regression models with time-varying covariates, we included interaction terms between class membership and fluid intake or norepinephrine dosing. The results showed that the main effect of fluid intake volume was positively associated with increased risk of death (HR 1.15; 95% CI 1.04–1.29; *p* = 0.009). There were significant interactions between class membership and fluid volume intake. While more fluid intake was associated with increased risk of death in class 4, more fluid intake was associated with reduced risk of death in class 2 (HR 0.81; 95% CI 0.69–0.95; *p* = 0.009). The effect of fluid volume on survival outcome is less prominent in other classes (Fig. 3A, C). Larger doses of norepinephrine were consistently associated with increased risk of mortality (HR 3.17; 95% CI 2.06–4.89; *p* < 0.001), and there was significant interaction between class 3 and norepinephrine dose (HR 0.28; 95% CI 0.14–0.58; *p* < 0.001; Fig. 3B, D).

### Optimal treatment strategy estimated by DTR

**Fig. 4:**
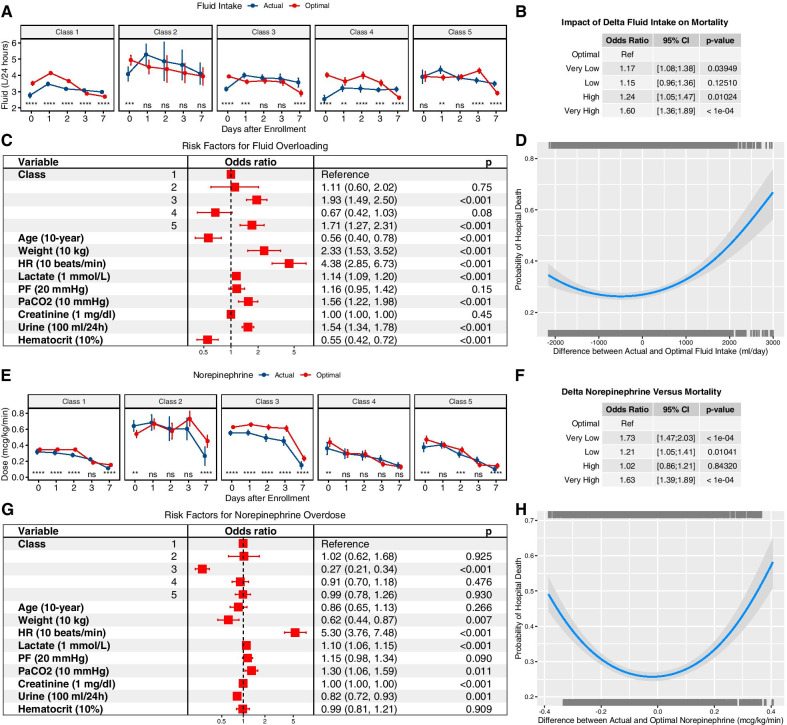
Optimal resuscitation strategy estimated by DTR. **A** Comparisons between actual and optimal fluid volume over days. The optimal fluid strategy is consistent with the concept of resuscitation/de-resuscitation model, especially in class 1 (baseline class) and class 3 (renal dysfunction class). However, class 3 showed earlier de-resuscitation than class 1 (day 1 vs. 3). More fluid could be given on day 0 for classes 1 to 4, indicating that initial resuscitation was usually inadequate in clinical practice. **B** Impact of delta fluid intake on mortality estimated by a logistic regression model fitting on validation set. Delta fluid intake was calculated as the difference between actual and optimal fluid intake at patient $\bullet$ day level and was categorized into five levels: very low (<−1000 mL), low (− 1000 to − 500 mL), optimal (− 500 to 500 mL), high (500 to 1000 mL) and very high (> 1000 mL). Odds ratio was reported by using optimal as reference. **C** Risk factors for fluid overloading. **D** DTR internal validation was performed by examining the relationship between delta fluid intake and mortality outcome. The trained DTR model estimated optimal fluid intake for each subject in the dataset from the Chinese multicenter cohort and a logistic regression model was trained by including a quadratic term for delta fluid intake. The parabolic curve indicates that the lowest mortality can be obtained at an optimal fluid strategy. **E** Comparisons between actual and optimal norepinephrine dose over days, stratified by class membership. The optimal dose was larger than the actual dose on day 0 for classes 1, 3, 4 and 5, indicating early initiation of norepinephrine could be beneficial for most classes. However, class 2 (critical class) showed lower/delayed initial dose would be beneficial. Combined with the result from fluid intake, it was deducible that initial large adequate fluid volume and delayed norepinephrine use were potentially beneficial for class 2. **F** Validation of the DTR model in the validation set by exploring the relative risk of mortality for different levels of delta norepinephrine dose. **G** Multivariable regression model exploring risk factors for norepinephrine overdose. **H** DTR model validation by examining the relationship between delta norepinephrine dose and mortality outcome

The optimal fluid volume and norepinephrine dosing were estimated using the DTR model. The predicted cluster labels by FMM were included in the DTR’s blip function. The actual and optimal treatment strategies were compared (Fig. 4A). The coefficients for the cluster labels 2, 3, 4 and 5 (one-hot encoding) in the blip function were 0.0575 (95% CI 0.0234–0.0876), 0.0453 (95% CI 0.0241–0.0761), − 0.0115 (95% CI: − 0.0761 to − 0.0034) and − 0.0126 (95% CI − 0.0616 to − 0.0021), respectively. Interestingly, the optimal fluid volume estimated by the DTR model showed a pattern with initially large volume over the first 2 days, followed by reduced volume requirement. This pattern is consistent with the concept of resuscitation and de-resuscitation in the management of septic shock. However, there is large difference among these classes. Class 2 showed longer period of resuscitation that significantly larger volume is required over the first 7 days than other classes. Norepinephrine was more likely to be overdosed on day 0 for class 2, while classes 1 and 3 were more likely to be underdosed (Fig. 4E). Analysis in the original dataset showed that smaller difference between actual and optimal dose resulted in a lower mortality risk (Fig. 4B, D, F, H).

### Risk factors for fluid and norepinephrine overdosing

To investigate risk factors for fluid overloading or norepinephrine overdosing, logistic regression models were built. The results showed that greater values of heart rate (OR for each 10 beats/min increase: 4.38; 95% CI 2.85–6.73; *p* < 0.001), class 3 (OR: 1.93; 95% CI 1.49–2.50; *p* < 0.001) and class 5 (OR 1.71; 95% CI 1.27–2.31; *p* < 0.001) were associated with increased risk of fluid overloading (Fig. 4C). Class 3 (OR 0.27; 95% CI 0.21–0.34; *p* < 0.001), body weight (OR for every 10-kg increase: 0.62; 95% CI 0.44–0.87; *p* = 0.007) and urine output (OR for every 100-mL increase: 0.82; 95% CI 0.72–0.93; *p* = 0.001) were associated with decreased risk of norepinephrine overdosing (Fig. 4G).

**Fig. 5:**
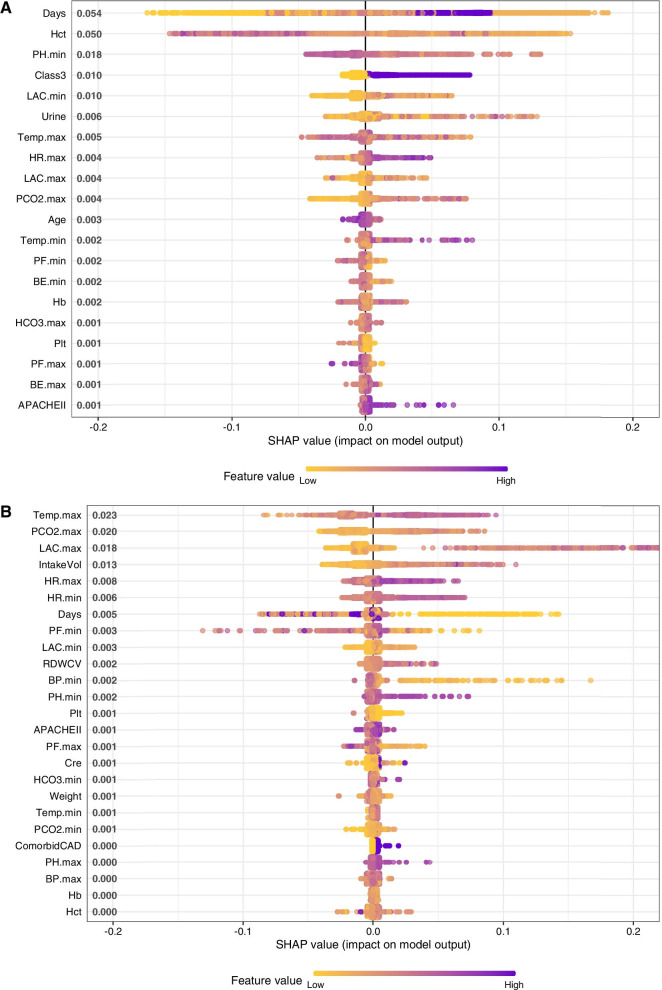
Risk factors for fluid and epinephrine overdosing explored using XGboost. Gradient color indicates the original value for that variable. Each point represents a row from the original dataset. **A** SHAP values of individual features in predicting the risk of fluid overloading (top 20 features are shown in the figure). ICU day was the most important variable predicting fluid overload. Patients in class 3 were more likely to receive fluid overload (the purple color indicates class 3 patients, and they contribute to increased risk of fluid overload as represented by the positive value on *x*-axis). **B** SHAP values of individual features in predicting the risk of norepinephrine overdosing (top 20 features are shown in the figure). Higher heart rate (purple color) was found to be associated with increased risk (positive SHAP value on *x*-axis) of norepinephrine overdosing

The logistic regression models require the assumption of a monotonic relationship between the dependent variable and the outcome, and this assumption is often violated in practice. Thus, we further used XGboost to identify risk factors for fluid overloading or norepinephrine overdosing [36]. The variable contribution to the model was explored by the SHapley Additive exPlanations (SHAP), in which the Shapley values calculate the importance of a feature by comparing what a model predicts with and without the feature [37]. ICU day was the most important variable predicting fluid overload. Patients in class 3 were more likely to receive fluid overload (the purple color indicates class 3 patients, and they contribute to increased risk of fluid overload as represented by the positive value on *x*-axis; Fig. 5A). SHAP values of individual features in predicting the risk of norepinephrine overdosing (top 20 features are shown in the figure). Higher heart rate (purple color) was found to be associated with increased risk (positive SHAP value on *x*-axis) of norepinephrine overdosing (Fig. 5B).

### External validation in eICU-CRD database

**Fig. 6:**
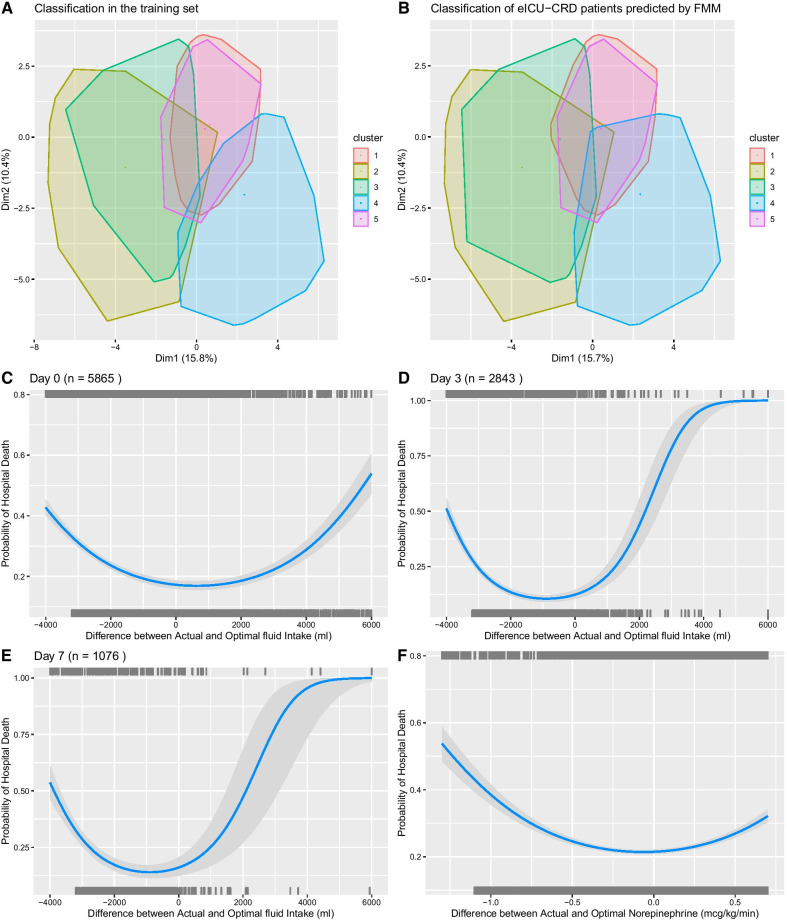
External validation in the eICU-CRD database. **A** Classification in the training dataset. The convex hulls of data points were assigned to the different clusters, and they were projected to two-dimensional space by principal component analysis. **B** Classification in the eICU-CRD dataset. The class membership of the new patients was determined by the highest probability predicted by the FMM. **C**–**E** Relationship between the difference between actual and optimal fluid volume and hospital mortality rate. The models were trained with quadratic terms for the difference between actual and optimal fluid volume. **F** The relationship between hospital mortality and the difference between actual and optimal norepinephrine dose

A total of 5856 patients with septic shock on ICU admission were identified from the eICU-CRD database. The mortality rate was 26.7% (1582/5865). The FMM model identified five classes of septic shock. The class membership in the eICU-CRD dataset was also predicted using the FMM method, which showed similar class distribution in the eICU-CRD (Fig. 6A, B). Furthermore, the DTR model was used to predict optimal dosing of fluid and norepinephrine on the eICU database. The differences between actual versus optimal dosing were calculated. The result showed that larger absolute value of the difference between actual and optimal doses was associated with increased hospital mortality (Fig. 6C–F).

## Discussion

The study identified five classes of septic shock that showed distinct clinical characteristics by using finite mixture modeling, which was further confirmed by *k*-means clustering. DTR model was employed to tailor individualized sequential treatment decisions, by estimating the optimal fluid volume and norepinephrine dosing. The five classes are clinically relevant in that (1) they are easily identifiable by routine clinical variables with stable prediction probability (minimum class membership probability > 0.80); (2) the optimal resuscitation strategy, which was confirmed in an independent dataset, differed across the five classes; (3) the transition from resuscitation to de-resuscitation phase should be different across classes so as to achieve a desirable clinical outcome; and (4) these classes showed differing risks of fluid overloading and norepinephrine overdosing. The system has important clinical implications that the heterogeneous septic shock population can be classified into subphenotypes and resuscitation strategy can be tailored by considering subphenotypes as well as their transitions across ICU days.

This study confirms previous findings that septic shock is a heterogeneous syndrome and can be classified into several stable subclasses [14, 19, 20]. The classification is stable in our multicenter cohort because both finite mixture modeling and *k*-means clustering arrive at the same number of classes. The minimum class membership probability is greater than 0.80. This study is different from previous studies that we focus solely on septic shock because we believe that this population requires urgent resuscitation and can benefit most from individualized treatment regimen. By using multicenter clinical data, Seymour CW and colleagues identified four types of sepsis, namely the $\alpha$, $\beta$, $\gamma$, $\delta$ phenotypes [21]. Septic shock is mostly in the $\delta$ phenotype, which is also associated with the highest mortality rate. From the perspective of immune responses, septic shock was classified into three subclasses in another study, but the small sample size prohibited finer classification [38]. Gårdlund and colleagues reported similar classes of septic shock. For example, the “uncomplicated septic shock” profile corresponds to class 5 (mild class) in our study, and the “severe septic shock” profile corresponds to class 2 (critical class) [19].

In the DTR framework [34,39, 40,41], the optimal sequential resuscitation rules were estimated. Days 0–3 were considered as resuscitation phase, and day 7 was considered as the de-resuscitation phase. Consistent with the concept of the “four D's” of fluid therapy [42, 43], our DTR model showed that larger fluid infusion and appropriate dosing of norepinephrine were usually required to achieve a better clinical outcome at an early phase, and less fluid infusion was beneficial at the late phase. Our study also showed that specific dosing strategies were different among classes. For example, the de-resuscitation phase began on day 3 for class 1 but began on day 1 for class 3 (renal failure class). Class 3 is at increased risk of fluid overload because the injured kidney is unable to effectively maintain fluid balance. Thus, patients in this class are more sensitive to fluid therapy, which is supported by rapid drop in optimal fluid volume from day 0 to day 1. Furthermore, class 3 is more likely to transition to other classes as it is the largest class at day 0, but the size decreases rapidly over time (Fig. 2C). The actual fluid volume was relatively low in class 4 (respiratory failure class), suggesting that physicians are aware of potential hazardous effect of fluid overloading for injured lungs [44–47].

There are numerous tools to evaluate fluid overload in clinical practice such as physical examination, chest radiography, natriuretic peptides, thoracic ultrasound and bioelectrical impedance analysis [48]. But these methods are inaccurate and variable across individuals. This study estimated the optimal fluid volume in the framework of DTR, which integrated many relevant clinical variables for model training, allowing for individualized fluid treatment strategy. The DTR models were also well validated in an independent dataset. Some interesting risk factors such as body weight, urine output and PaCO_2_ were identified for fluid overloading. The body weight may not be a good marker to determine fluid dose because fluid retention is common in critically ill patients. Increased body weight is a sign of fluid overload, but in reality, physicians may prescribe too much fluid based on the formula for calculating fluid requirement. Furthermore, patients with high PaCO_2_ are more likely to have fluid overload because PaCO_2_ retention can be the result of severe ARDS and protective ventilation, in which the optimal fluid is usually conservative in order to improve clinical outcomes.

Norepinephrine is usually required to maintain blood pressure after fluid infusion. However, the timing and dosing of norepinephrine are largely based on subjective judgment. As compared to the real clinical practice in our participating hospitals, less norepinephrine and more fluids can be given to class 2 patients on day 0, in order to achieve a better clinical outcome. This is consistent with some observations that treatment strategy with more fluid volume and lower vasopressor dose at 0–6 h is associated with improved mortality outcome [49, 50]. Patients in class 2 are characterized by profound hypotension and poor tissue perfusion. The use of norepinephrine may further reduce tissue perfusion when fluid infusion is inadequate. It is interesting to note that the norepinephrine dosing is similar between actual and optimal strategy on day 1, which supports the well-accepted concept that adequate fluid infusion should be given before considering vasopressors. Results from observational studies showed that early norepinephrine use is associated with improved mortality outcome [51–54], which is further supported by a small RCT [55]. This is not contradictory to our results. The actual norepinephrine is underdosed on days 0 and 1 in classes 1 and 3 in our study. In other words, early larger dose of norepinephrine in these two classes could help improve outcomes. Since classes 1 and 3 comprise the majority of septic shock population, it is not surprising that the undifferentiated septic shock patients enrolled in those trials can benefit from early vasopressor use.

There are several limitations that must be acknowledged in the study. Although we tried to classify septic shock patients into five subclasses, the granularity may not be high enough to fully implement the individualized resuscitation strategy. Sepsis is a highly heterogeneous and dynamic syndrome, and it is likely that different patients within each of the clusters will require different resuscitation regimen. However, higher granularity means the requirement of large sample size and makes the explainability of the model more challenging. We need to strike a balance between granularity, sample size requirement and explainability. Secondly, there could be residual confounding effect in the Cox regression and the DTR models due to the observational nature of the study design. For example, the study showed that larger doses of norepinephrine were consistently associated with increased risk of mortality, which could be explained by the fact that patients requiring larger dose of norepinephrine were more critically ill. Thirdly, the minimum number of patients was predefined to ensure each cluster contained clinically meaningful size, so that optimal treatment strategy could be explored within each cluster. However, it is possible that by forcing patients into larger/fewer clusters, patients with potentially different treatment responses are being lumped together. This is actually a trade-off between high granularity and model explainability. Finally, it is ideal to explore the fluid and norepinephrine dosing in the same model. However, the unique combinations of treatments can result in large number of interventions, which is not allowed with limited sample size.

## Conclusions

In conclusion, our study identified five distinct classes of septic shock. These classes are useful for guiding individualized resuscitation strategy and designing future trials involving septic shock. By comparing actual and optimal treatment strategy, the risk factors for fluid overload and norepinephrine overdosing were explored. Our results support the previous finding that early initiation of norepinephrine is beneficial in the majority of septic shock patients, but also complement this finding that delayed norepinephrine is beneficial for a subclass of septic shock.

## Supplementary Information

**Additional file 1.** Supplemental Digital Content for Individualized resuscitation strategy forseptic shock formalized by finite mixture modeling and dynamic treatment regimen.

**Additional file 2.** R code for data analysis.

## Data Availability

Data are available upon reasonable request and with the approval from the Ministry of Science and Technology of the People’s Republic of China.

## Abbreviations

DTR: Dynamic treatment regime
eICU-CRD: EICU Collaborative Research Database
ARDS: Acute respiratory distress syndrome
SOFA: Sequential Organ Failure Assessment
ICU: Intensive care unit
RCT: Randomized controlled trial
HCT: Hematocrit
AUC: Area under the curve
CI: Confidence interval
SHAP: SHapley Additive exPlanations
OR: Odds ratio
