# Supplementary material for: Individualized resuscitation strategy for septic shock formalized by finite mixture modeling and dynamic treatment regimen
Source: Crit Care. 2021 Jul 12;25:243. doi: 10.1186/s13054-021-03682-7 (PMC8273991; doi:10.1186/s13054-021-03682-7)
Supplement: Supplementary file 1 — Additional file 1. Supplemental Digital Content for Individualized resuscitation strategy forseptic shock formalized by finite mixture modeling and dynamic treatment regimen. [file 13054_2021_3682_MOESM1_ESM.docx]

Supplemental Digital Content for Individualized resuscitation strategy for septic shock formalized by finite mixture modeling and dynamic treatment regimen

Penglin Ma (MD)#

Department of Critical Care Medicine, the 8th Medical Center of Chinese PLA General Hospital, 100091, Beijing, P. R. China.

Jingtao Liu (MD)#

Department of Critical Care Medicine, the 8th Medical Center of Chinese PLA General Hospital, 100091, Beijing, P. R. China. Email: [ljt309@sohu.com](mailto:ljt309@sohu.com)

Feng Shen

Department of Intensive Care Unit, Guizhou Medical University Affiliated Hospital;

Xuelian Liao

Department of Critical Care Medcine, West China Hospital of Sichuan University

Ming Xiu

Department of Intensive Care Unit, The First Hospital of Jilin University

Heling Zhao

Department of Critical Care Medicine,Hebei General Hospital

Mingyan Zhao

Department of Critical Care Medicine, The First Affiliated Hospital of Harbin Medical University

Jing Xie

General Intensive Care Unit Department, The First Aﬃliated Hospital of Fujian Medical University

Peng Wang

Department of Critical Care Medicine, Fu Xing Hospital, Capital Medical University

Man Huang

General Intensive Care Unit, Second Affiliated Hospital of Zhejiang University

Tong Li

Department of Critical Care Medicine, Beijing Tongren Hospital，Capital Medical University

Meili Duan

Department of Critical Care Medicine, Beijing Friendship Hospital，Capital Medical University.

Kejian Qian

Department of Critical Care Medicine, The first Affiliated Hospital of Nanchang University

Yue Peng

Department of Critical Care Medicine, The third Xiangya hospital, Central South University

Feihu Zhou

Department of Critical Care Medicine, Chinese PLA General Hospital

Xin Xin

Surgical Intensive Care Unit, Beijing Chao-Yang Hospital, Capital Medical University

Xianyao Wan

The First Affiliated Hospital of Dalian Medical University

ZongYu Wang

Department of Intensive Care, Peking University Third Hospital

Shusheng Li

Department of Emergency, Tongji Hospital, Tongji Medical College, Huazhong University of Science and Technology

Jianwei Han

Department of Critical Care Medicine, the 8th Medical Center of Chinese PLA General Hospital

Zhenliang Li

Department of Critical Care，Beijing PingGu Hospital，Capital Medical University

Guolei Ding

Intensive Care Unit,The hospital of Shunyi District

Qun Deng

Department of Critical Care Medicine, the 4th Medical Center of Chinese PLA General Hospital

Jicheng Zhang

Department of Critical Care Medicine，Shandong Provincial Hospital Affiliated to Shandong First Medical University

Yue Zhu

Department of Critical Care，Beijing Luhe Hospital，Capital Medical University

Wenjing Ma

Department of Critical Care，Beijing Miyun Hospital

Jingwen Wang

Intensive Care Unit，Beijing Changping District Hospital

Yan Kang

Department of Critical Care Medicine, West China Hospital of Sichuan University. Email: [kangyan@scu.edu.cn](mailto:kangyan@scu.edu.cn)

Zhongheng Zhang (MD)*

Department of Emergency Medicine, Sir Run Run Shaw Hospital, Zhejiang University School of Medicine, Hangzhou, 310016, China. Email: [zh_zhang1984@zju.edu.cn](mailto:zh_zhang1984@zju.edu.cn)

# PM and JL contributed equally to this work and should be considered as co-first author.

Correspondence to: Zhongheng Zhang (MD)*

Department of Emergency Medicine, Sir Run Run Shaw Hospital, Zhejiang University School of Medicine, Hangzhou, 310016, China. Email: [zh_zhang1984@zju.edu.cn](mailto:zh_zhang1984@zju.edu.cn)

Table of Contents

[Methods 1](#_Toc71030395)

[Finite mixture modeling 1](#_Toc71030396)

[Dynamic treatment regimen modeling 2](#_Toc71030397)

[DTR model validation 4](#_Toc71030398)

[Risk factors for fluid overloading and norepinephrine overdosing 4](#_Toc71030399)

[Reference: 5](#_Toc71030400)

# Methods

## Finite mixture modeling

The classes of septic shock were explored by using finite mixture modeling with feature variables being normalized. Normalization was performed by centering at mean value and divided by the standard deviation for each feature. Missing values were imputed with mean values. Correlation between feature variables were examined using Pearson’s correlation analysis. We removed highly correlated variables by domain knowledge. Candidate variables representing several key pathophysiological domains were included for FMM, such as baseline demographics (age, weight), disease severity (APACHE II), vital signs (SBP, DBP, HR, temperature, RR), tissue perfusion (lactate), internal environment (BE, pH, HCO_3_), respiration (PaCO_2_, PaO_2_, PF) and inflammatory responses (CRP, RDWCV), hematology (platelet) and renal function (urine output, creatinine). PaO_2_, CRP and DBP were removed due to their correlation with other variables with correlation coefficient > 0.7. The FMM was fit to the combined dataset of feature vectors from all patients across day 0, 1, 2, 3 and 7, while allowing class transition across ICU days. The best number of classes were determined by both statistics and clinical importance. The best number of classes were determined by both fit statistics and clinical importance. Lower values of AIC and SABIC, higher values of entropy were considered as better model fit. Bootstrap likelihood ratio test was performed to compare whether k-class model was better than (k-1)-class model(2, 3). The minimum number of patients should be over 4%. The minimum probability of assigning to one class should be over 0.8, otherwise, the class membership is considered as unstable.

The best number of classes was also confirmed by the k-means clustering analysis(4). Statistics such as Cubic Clustering Criterion (CCC), Calinski and Harabasz (CH) index, Davies and Bouldin (DB) index, Hartigan, Krzanowski and Lai (KL) index, Marriot, Rubin, TraceW were reported. The characteristics of each of the index are shown in the following table.

Table 1 Characteristics of each index for determing the best number of clusters

| Index | Description |
| --- | --- |
| Cubic Clustering Criterion (CCC) | The CCC is based on the assumption that clusters obtained from a uniform distribution on a hyperbox are hypercubes of the same size. The hypercube assumption is obviously false in most cases, but is generally conservative unless the number of clusters is very large in two or more dimensions. The maximum value of the index is used to indicate the optimal number of clusters in the data set. |
| Calinski and Harabasz (CH) index | The CH Index (also known as Variance ratio criterion) is a measure of how similar an object is to its own cluster (cohesion) compared to other clusters (separation). Here cohesion is estimated based on the distances from the data points in a cluster to its cluster centroid and separation is based on the distance of the cluster centroids from the global centroid. |
| Davies and Bouldin (DB) index | The intuition behind Davies-Bouldin index is the ratio between the within cluster distances and the between cluster distances and computing the average overall the clusters. It is therefore relatively simple to compute, bounded – 0 to 1, lower score is better. However, since it measures the distance between clusters’ centroids it is restricted to using Euclidean distance function. |
| Hartigan index | The maximum difference between hierarchy levels is taken as indicating the correct number of clusters in the data |
| Krzanowski and Lai (KL) index | It is based on the criteria for determining the number of groups in a data set using sumof-squares clustering |
| Marriot | The maximum difference between successive levels is used to determine the best partition level |
| Rubin | It is a criterion based on the ratio of the determinant of the total sum of squares and cross products matrix to the determinant of the pooled within cluster matrix. The minimum value of second differences between levels is used to select the optimal number of clusters. |
| TraceW | This index has been one of the most popular indices suggested for use in clustering context. Given that the criterion increases monotonically with solutions containing fewer clusters, the maximum of the second differences scores are used to determine the number of clusters in the data. |

## Dynamic treatment regimen modeling

A dynamic treatment regimen is a set of rules for choosing effective treatments for individual patients. Treatment choices made for a particular patient under a dynamic regime are based on that individual's characteristics and history, with the goal of optimizing his or her final clinical outcome after a sequential decision rule. A dynamic treatment regime is analogous to a policy in the field of reinforcement learning, and analogous to a controller in control theory. Briefly, the idea of DTR is to estimate optimal treatment strategy across multiple treatment stages, so that the final clinical outcome can be optimized (25). In this study, the treatments are continuous variables including fluid volume intake and norepinephrine or equivalent dosing. The regression-based method was used for estimating the optimal dosing strategy across day 0, 1, 2, 3, and 7 after ICU admission. More specifically, the mortality outcome $E\left( Y|x,a \right)$ was modelled in terms of treatment free model $f\left( x^{\beta}; \beta\right)$ and a blip function $\gamma\left( x^{\psi}, a; \psi\right)$: $E\left( Y|x,a \right)=f\left( x^{\beta}; \beta\right)+\gamma\left( x^{\psi}, a; \psi\right)$, where $x^{\beta}$ and $x^{\psi}$ are subsets of observed covariates vector $\mathbf{x}$. The blip function is parameterized in terms of $\psi$ and characterizes the treatment effect. The blip function characterizes the main treatment effect as well as its interaction(s) with patient-specific covariates. The variables included in the blip model component are also referred to as tailoring covariates as they allow for the identification of patient-specific treatment interactions and ultimately lead to the identification of the optimal personalized treatment decision. DTR model also requires specification of a treatment model, which is a propensity score for receiving treatment: $\pi\left( a|x \right)=f_{A|x}\left( a|x \right)$. The goal of parameter estimation is to optimize the final outcome $Y$ in a sequential manner. The estimation was performed by dynamic weighted ordinary least squares(26).

Two DTR models were trained for fluid intake and norepinephrine dosing, respectively. Covariates interacting with the treatment (fluid intake or norepinephrine dose) included heart rate, blood pressure, temperature, class, urine output, hematocrit, PF, pH, lactate and daily output. The specification of the blip function can be formalized with the following pseudocode:

Xpsi1<-list(

~HR.max_0+BP.min_0+Temp.max_0+Class_0+Urine_0+Hct_0+

PF.min_0+PH.min_0+LAC.max_0+OutputVol_0,

~HR.max_1+BP.min_1+Temp.max_1+Class_1+Urine_1+Hct_1+

PF.min_1+PH.min_1+LAC.max_1+OutputVol_1,

~HR.max_2+BP.min_2+Temp.max_2+Class_2+Urine_2+Hct_2+

PF.min_2+PH.min_2+LAC.max_2+OutputVol_2,

~HR.max_3+BP.min_3+Temp.max_3+Class_3+Urine_3+Hct_3+

PF.min_3+PH.min_3+LAC.max_3+OutputVol_3,

~HR.max_7+BP.min_7+Temp.max_7+Class_7+Urine_7+Hct_7+

PF.min_7+PH.min_7+LAC.max_7+OutputVol_7

)

The treatment model is a list of formula objects specifying the treatment model at each stage in order. The treatment variable should be included as the dependent variable. We specified that body weight, blood pressure, output volume, heart rate and body temperature can influence the actual fluid volume intake. For example, physicians may give more fluid to a subject with high body temperature, low blood pressure and high heart rate. The fluid infusion is also influenced by the fluid output. The treatment model can be specified with the following pseudocode:

treat.mod <- list(

IntakeVol_0~Weight+HR.max_0+BP.min_0+Temp.max_0+OutputVol_0,

IntakeVol_1~Weight+HR.max_1+BP.min_1+Temp.max_1+OutputVol_1,

IntakeVol_2~Weight+HR.max_2+BP.min_2+Temp.max_2+OutputVol_2,

IntakeVol_3~Weight+HR.max_3+BP.min_3+Temp.max_3+OutputVol_3,

IntakeVol_7~Weight+HR.max_7+BP.min_7+Temp.max_7+OutputVol_7

)

Treatment free model is a list of formula objects specifying covariates of a (linear) treatment-free model for each stage in order. No dependent variable should be specified. Variables in the treatment free model are those that can have impact on mortality but did not have direct impact on the determination of fluid volume. We choose APACHE II on day 0, age, gender, RDWCV, creatinine, hematocrit and lactate for the treatment free model.

## tf.mod<-list(~APACHEII+Age+Gender+RDWCV_0+Cre_0+Hct_0+LAC.max_0,

## ~ Age+Gender+RDWCV_1+Cre_1+Hct_1+LAC.max_1,

## ~ Age+Gender+RDWCV_2+Cre_2+Hct_2+LAC.max_2,

## ~ Age+Gender+RDWCV_3+Cre_3+Hct_3+LAC.max_3,

## ~ Age+Gender+RDWCV_7+Cre_7+Hct_7+LAC.max_7)

## DTR model validation

DTR model validation was performed in an independent validation set (eICU-CRD). The DTR model was trained in the training set and validated in the validation set. The results of the DTR model would return individualized optimal dosing strategy for both fluid volume and norepinephrine dosing across day 0, 1, 2, 3 and 7. Each of the patients in the validation set can have an actual and the optimal fluid volume and norepinephrine dose (i.e., the optimal value was estimated with the DTR model trained in the training set). Then, the actual treatment strategy was compared to the optimal treatment strategy. Delta fluid intake was calculated as the difference between actual and optimal fluid intake. Delta fluid intake was categorized into five levels: very low (< -1000 ml), low (-1000 to -500 ml), optimal (-500 to 500 ml), high (500 to 1000 ml), and very high (> 1000 ml). Relative mortality risk of each level was explored using logistic regression model and odds ratio was reported by using the optimal level as reference. Furthermore, the delta fluid intake was modeled with quadratic functional form in a logistic regression model. This functional form allows identification of a nadir delta fluid intake associated with lowest mortality risk in a parabolic curve. The DTR model was regarded as well fitted if the lowest mortality risk was found around delta fluid intake = 0. The same validation method was applied to the DTR model for norepinephrine dosing.

## Risk factors for fluid overloading and norepinephrine overdosing

Fluid overloading was defined as those receiving > 1000 ml/day than the optimal volume, and norepinephrine overdosing was those receiving > 0.1 mcg/kg/min than the optimal dose. Risk factors for fluid and norepinephrine overdosing were explored by using stepwise backward elimination and foreword selection logistic regression models with AIC criteria.

# Reference:

1. García CB, García J, López Martín MM, Salmerón R. Collinearity: revisiting the variance inflation factor in ridge regression. *Journal of Applied Statistics*, 2nd ed. 2015;42:648–661.

2. Nasserinejad K, van Rosmalen J, de Kort W, Lesaffre E. Comparison of Criteria for Choosing the Number of Classes in Bayesian Finite Mixture Models. In: Tran US, editor. *PLoS ONE* 2017;12:e0168838.

3. Nylund KL, Asparouhov T, Muthén BO. Deciding on the number of classes in latent class analysis and growth mixture modeling: A Monte Carlo simulation study. *Structural Equation Modeling*, 4 ed. 2007;14:535–569.

4. Charrad M, Ghazzali N, Boiteau V, Niknafs A. NbClust: An RPackage for Determining the Relevant Number of Clusters in a Data Set. *Journal of Statistical Software* 2014;61:.
