# Supplementary material for: Individualized resuscitation strategy for septic shock formalized by finite mixture modeling and dynamic treatment regimen
Source: Crit Care. 2021 Jul 12;25:243. doi: 10.1186/s13054-021-03682-7 (PMC8273991; doi:10.1186/s13054-021-03682-7)
Supplement: Supplementary file 2 — Additional file 2. R code for data analysis. [file 13054_2021_3682_MOESM2_ESM.pdf]

````
---
title: "DynFluidSepsis"
author: "Zhongheng Zhang"
date: "11/9/2020"
output: html_document
---

```{r setup, include=FALSE}
knitr::opts_chunk$set(echo = TRUE)
library(readxl)
library(tidyverse)
library(lubridate)
library(factoextra)
library(DataExplorer)
library(missForest)
library(cluster)
library(ggalluvial)
library(reinforcelearn)
library(ggpubr)
library(cowplot)
library(gridExtra)
library(ggsci)
library(CBCgrps)
library(Publish)
library(tidyLPA)
```


#Data Management
```{r DataManageemnt}
dtPtBase <- read_xlsx("dat.xlsx",sheet = "基础信息") %>%
  arrange(医院,姓名,入ICU时间,月...21,日...22) %>%
  distinct(医院,姓名,入ICU时间,月...21,日...22,.keep_all = T) %>%
  mutate(name=姓名) %>%
  group_by(医院,姓名) %>%
  group_modify(~{
    if(nrow(.x)>1){
      .x$name <- paste(.x$name,c("",1:(nrow(.x)-1)),sep = "")
    }
    return(.x)
  }) %>% ungroup() %>%
  mutate(
    sepsis诊断时间=if_else(is.na(sepsis诊断时间),
                       入ICU时间,
                       sepsis诊断时间),
    月...16=if_else(is.na(月...16),月...21,月...16),
    日...17=if_else(is.na(日...17),日...22,日...17),
    时...18=if_else(is.na(时...18),时...23,时...18),
    分...19=if_else(is.na(分...19),分...24,分...19)
    ) %>%
  mutate(
    时...18=if_else(is.na(时...18),"0",时...18),
    时...18=if_else(parse_double(时...18)>23,
````
````

```
                  as.character(parse_double(时...18)/100),时...18),
    分...19=if_else(is.na(分...19),"0",分...19)
  ) %>%
  filter(!is.na(sepsis诊断时间)) %>%
  mutate(
    Onset_time=paste(
      paste(sepsis诊断时间,月...16,日...17,sep = "-"),
      paste(时...18,分...19,"0",sep = ":")
      ),
    ICUin_time=paste(
      paste(入ICU时间,月...21,日...22,sep = "-"),
      paste(时...23,分...24,"0",sep = ":")
    ),
    MVstart_time=paste(
      paste(MV时间,月...27,日...28,sep = "-"),
      paste(时...29,"0","0",sep = ":")
    ),
    MVstop_time = paste(
      paste(MV结束,月...31,日...32,sep = "-"),
      paste(时...33,"0","0",sep = ":")
    ),
    Mort=if_else(is.na(`28天死亡=1`)|`28天死亡=1`==0,0,2-`28天死亡=1`)
    ) %>%
  mutate(across(ends_with("_time"),as_datetime)) %>%
  mutate(
    Onset_time = if_else(is.na(Onset_time),ICUin_time,Onset_time),
    ICUin_time = if_else(is.na(ICUin_time),Onset_time,ICUin_time)
  )%>%
  select(!contains(c("...","姓名","28天死亡")))
dtCirc <- read_xlsx("dat.xlsx",sheet = 2) %>%
  arrange(医院,姓名,`血管活性药物停用时间（年）`,
          `血管活性药物停用时间（月）`,
          `血管活性药物停用时间（日）`) %>%
  distinct(医院,姓名,`血管活性药物停用时间（年）`,
          `血管活性药物停用时间（月）`,
          `血管活性药物停用时间（日）`,.keep_all = T) %>%
  mutate(name=姓名) %>%
  group_by(医院,姓名) %>%
  group_modify(~{
    if(nrow(.x)>1){
      .x$name <- paste(.x$name,c("",1:(nrow(.x)-1)),sep = "")
    }
    return(.x)
  }) %>% ungroup() %>%
  rename_with(.col=contains("最"),~paste(.x,parse_number(.x),sep = "_"))
%>%
  rename_with(~str_remove(.x,"[[:digit:]]")) %>%
  rename_with(~str_remove(.x,"d")) %>%
  rename_with(~str_remove(.x,"剂量")) %>%
  rename_with(~str_replace(
    .x,"肾上腺最（ug/kg/min）_1",
```

```
    "肾上腺最低（ug/kg/min）_1")
    ) %>%
  mutate(across(!c(医院,姓名,住院号,name)&where(is.character),
              ~ parse_double(.x))) %>%
  dplyr::select(contains(c("医院","name","_"))) %>%
  pivot_longer(
    cols=contains("_"),
    names_to=c(".value", "days"),
    names_sep = "_"
    ) %>%
  rename(
    Norepi.max=`去甲最高（ug/kg/min）`,
    Norepi.min=`去甲最低（ug/kg/min）`,
    Dopa.max=`多巴胺最高（ug/kg/min）`,
    Dopa.min=`多巴胺最低（ug/kg/min）`,
    Epinephrin.max=`肾上腺最高（ug/kg/min）`,
    Epinephrin.min=`肾上腺最低（ug/kg/min）`,
    Dobuta.max=`多巴酚最高（ug/kg/min）`,
    Dobuta.min=`多巴酚最低（ug/kg/min）`,
    Vasopressor.max=`垂体最高（u/h）`,
    Vasopressor.min=`垂体最低（u/h）`,
    HR.max=`最快心率`,
    HR.min=`最慢心率`,
    BP.max=最高血压,BP.min=最低血压,Temp.max=最高体温,
    Temp.min=最低体温,
    CVP.max=最高CVP,CVP.min=最低CVP,
    ScvO2.max=最高scvo,ScvO2.min=最低scvo,
    BNP.max=最高BNP,BNP.min=最低BNP
         ) %>%
  mutate(Norepi=if_else(is.na(Norepi.max)&is.na(Norepi.min),0,1),
         Dopamine=if_else(is.na(Dopa.max)&is.na(Dopa.min),0,1)
         ) %>%
  mutate(across(Norepi.max:Vasopressor.min,
              ~if_else(is.na(.x),0,.x)),
         NorepiEq=pmax(Norepi.max,Norepi.min)+
           pmax(Epinephrin.max,Epinephrin.min)+
           pmax(Dopa.max,Dopa.min)/100+
           pmax(Vasopressor.max,Vasopressor.min)*2.5/60
         )
dtlab <- read_xlsx("dat.xlsx",sheet = 3) %>%
  rename(姓名=icu1,住院号=n1aa2aa) %>%
  arrange(医院,姓名) %>%
  mutate(name=姓名) %>%
  group_by(医院,姓名) %>%
  group_modify(~{
    if(nrow(.x)>1){
      .x$name <- paste(.x$name,c("",1:(nrow(.x)-1)),sep = "")
    }
    return(.x)
  }) %>% ungroup() %>%
  rename_with(.col=`0d PHmax`:daaddimer4,
```

```
              ~paste(.x,rep(c(0,1,2,3,7),length(.x)/5),sep = "_")) %>%
  rename_with(~str_remove(.x,"[:digit:](d| Day)")) %>%
  rename_with(~str_remove(.x," ")) %>%
  rename_with(
    .col=contains(c("dimer","Dimer")),
    ~paste("DDimer",c(0:3,7),sep = "_")
    ) %>%
  mutate(across(!c(医院,姓名,住院号,name)&where(is.character),
               ~ parse_double(.x))) %>%
  pivot_longer(
    cols=contains("_"),
    names_to=c(".value", "days"),
    names_sep = "_"
    ) %>%
  select(!matches("姓名")) %>%
  rename(PFmax=氧合指数max,
         PFmin=氧合指数mini) %>%
  rename_with(~str_remove(.x,"\\(.*\\)|\\（.*\\）")) %>%
  rename_with(~str_replace(.x,"min",".min")) %>%
  rename_with(~str_replace(.x,"max",".max"))
dtfluid <- read_xlsx("dat.xlsx",sheet = 4) %>%
  select(!matches("其他")&!starts_with("ae")) %>%
  mutate(name=姓名) %>%
  group_by(医院,姓名) %>%
  group_modify(~{
    if(nrow(.x)>1){
      .x$name <- paste(.x$name,c("",1:(nrow(.x)-1)),sep = "")
    }
    return(.x)
  }) %>% ungroup() %>%
  rename_with(.col=matches("[[:digit:]]d"),
              ~paste(.x,parse_number(.x),sep = "_")) %>%
  rename_with(~str_remove(.x,"[:digit:]d")) %>%
  rename_with(~str_remove(.x," ")) %>%
  mutate(across(!c(医院,姓名,住院号,name)&where(is.character),
               ~ parse_double(.x))) %>%
  select(医院,name,住院号,contains("_")) %>%
  rename_with(.col=matches("^[[:digit:]]"),
              ~paste("Fld",.x,sep = "")) %>%
  pivot_longer(
    cols=contains("_")&!matches("小时数_0"),
    names_to=c(".value", "days"),
    names_sep = "_"
    )
dtfinal <- dtPtBase %>%
  left_join(dtCirc,by=c("医院","name")) %>%
  left_join(dtlab,by=c("医院","name","days")) %>%
  left_join(dtfluid,by=c("医院","name","days")) %>%
  group_by(医院,name) %>%
    group_modify(~ {
      .x$N <- nrow(.x)
      return(.x)
```

```r
    }) %>% ungroup() %>%
  filter(N==5) %>%
  dplyr::select(!contains(c(".y","住院号"))) %>%
  rename_with(.cols = ends_with(".x"),~str_remove(.x,".x")) %>%
  rename_with(~str_replace(.x,"-","")) %>%
  rename_with(~str_replace(.x," ","")) %>%
  rename(Hospital=医院,Gender=`性别男=1`,Weight=体重,Height=身高,
         Age=年龄,Diagnosis=诊断,Diagnosis1=n2,Diagnosis2=n3,
         Comorbid=`伴随疾病糖尿病=1，高血压=2，CRF=3，冠心病=4`,
         SiteInfect=感染部位,
         Type=`是否手术择期=1，急诊=2，否=3`,
         SIRS=`sirs=1`,
         Hospital_LOS=总住院天数,
         ICU_LOS=ICU住院天数,
         IntakeVol=总入量,
         APACHEII = `apache II`,
         OutputVol=总出量,Urine=尿量,
         MV=`MV=1`
         ) %>%
  mutate(PCO2.max=coalesce(PCO2.max,PCO2.min),
         PCO2.min=coalesce(PCO2.min,PCO2.max),
         HR.max=coalesce(HR.max,HR.min),
         HR.min=coalesce(HR.min,HR.max),
         BP.max=coalesce(BP.max,BP.min),
         BP.min=coalesce(BP.min,BP.max),
         Temp.max=coalesce(Temp.max,Temp.min),
         Temp.min=coalesce(Temp.min,Temp.max),
         CVP.max=coalesce(CVP.max,CVP.min),
         CVP.min=coalesce(CVP.min,CVP.max),
         ScvO2.max=coalesce(ScvO2.max,ScvO2.min),
         ScvO2.min=coalesce(ScvO2.min,ScvO2.max),
         BNP.max=coalesce(BNP.max,BNP.min),
         BNP.min=coalesce(BNP.min,BNP.max),
         PH.max=coalesce(PH.max,PH.min),
         PH.min=coalesce(PH.min,PH.max),
         HCO3.max=coalesce(HCO3.max,HCO3.min),
         HCO3.min=coalesce(HCO3.min,HCO3.max),
         BE.max=coalesce(BE.max,BE.min),
         BE.min=coalesce(BE.min,BE.max),
         LAC.max=coalesce(LAC.max,LAC.min),
         LAC.min=coalesce(LAC.min,LAC.max),
         PF.max=coalesce(PF.max,PF.min),
         PF.min=coalesce(PF.min,PF.max),
         SiteInfect=if_else(is.na(SiteInfect),"胸部",SiteInfect)
         ) %>%
  mutate(
    BP.min=ifelse(BP.min>=300|BP.min<=20,mean(BP.min,na.rm = T),BP.min),
    BP.max=ifelse(BP.max>=300|BP.max<=20,mean(BP.max,na.rm = T),BP.max),
    Cre= ifelse(Cre<20,Cre*88.4,Cre),
    HCO3.max = ifelse(HCO3.max<=0,-HCO3.max,HCO3.max),
    HCO3.min = ifelse(HCO3.min<=0,-HCO3.min,HCO3.min),
    Hct = ifelse(Hct>80|Hct<10,mean(Hct,na.rm = T),Hct),
```

```
    HR.max = ifelse(HR.max>200|HR.max<20,mean(HR.max,na.rm = T),HR.max),
    HR.min = ifelse(HR.min>200|HR.min<20,mean(HR.min,na.rm = T),HR.min),
    LAC.max=ifelse(LAC.max>30|LAC.max<0,mean(LAC.max,na.rm = T),LAC.max),
    LAC.min=ifelse(LAC.min>30|LAC.min<0,mean(LAC.min,na.rm = T),LAC.min),
    PCO2.max=ifelse(PCO2.max>150|PCO2.max<20,mean(PCO2.max,na.rm =
T),PCO2.max),
    PCO2.min=ifelse(PCO2.min>150|PCO2.min<20,mean(PCO2.min,na.rm =
T),PCO2.min),
    PF.min=ifelse(PF.min>600|PF.min<20,mean(PF.min,na.rm = T),PF.min),
    PF.max=ifelse(PF.max>600|PF.max<20,mean(PF.max,na.rm = T),PF.max),
    PH.min = if_else(PH.min<6.9,6.9,
                     if_else(PH.min>70,PH.min/10,
                             if_else(PH.min>7.9,mean(PH.min,na.rm =
T),PH.min))),
    Plt = ifelse(Plt>1000,1000,Plt),
    RDWCV = ifelse(RDWCV>50,RDWCV/10,RDWCV),
    Hb = ifelse(Hb>300|Hb<30,median(Hb,na.rm = T),Hb),
    Temp.max = ifelse(Temp.max<34|Temp.max>42,mean(Temp.max,na.rm =
T),Temp.max),
    Temp.min = ifelse(Temp.min<34|Temp.min>42,mean(Temp.min,na.rm =
T),Temp.min),
    Norepi.max = if_else(Norepi.max>3,3,Norepi.max),
    Dopa.max = if_else(Dopa.max>20,20,Dopa.max),
    MV=if_else(is.na(MV),0,2-MV),
    Urine=if_else(Urine>30000,median(Urine,na.rm = T),Urine),
    IntakeVol=if_else(IntakeVol>20000|IntakeVol<500,
                      median(IntakeVol,na.rm = T),IntakeVol),
    OutputVol=if_else(OutputVol>20000|OutputVol<50,
                      median(OutputVol,na.rm = T),OutputVol),
    Comorbid=if_else(
      is.na(Comorbid),"None",
      if_else(
        Comorbid==1,"Diabetes",
        if_else(Comorbid==2,"Hypertension",
                if_else(Comorbid==3,"CRF","CAD")))),
    SiteInfect=recode(SiteInfect,腹腔="Abdomen",胸部="Thorax",腹部="Abdomen",
                      胸腔="Thorax",颅内="Brain",血流感染="Blood",
                      皮肤软组织="SoftTissue",泌尿系="UTI",
                      颅脑 ="Brain"),
    Type=if_else(
      is.na(Type),"NonOperation",
      if_else(Type==2,"Emergency",
              if_else(Type==1,"Elective","NonOperation"))
    ),
    days=parse_number(as.character(days))
    ) %>%
  mutate(across(where(is.character),as_factor)) %>%
  mutate(Comorbid=fct_relevel(Comorbid,"None")) %>%
  dplyr::select(-c(Diagnosis1, Diagnosis2, ae1,sepsis诊断时间,入ICU时间,
           MV时间,MV结束,`体温=1`:年,最高,最低,N,)) %>%
  dplyr::select(where(~sum(is.na(.x))/length(.x)<0.25)&
           !where(is.POSIXct)) %>%
```

````
  group_by(Hospital,name) %>%
  fill(everything(),.direction = "updown") %>% ungroup() %>%
  mutate(across(.col=where(is.numeric),~if_else(is.na(.x),median(.x,na.rm =
T),.x))) %>%
  mutate(
    Vasopressor = dplyr::select(., Norepi.max) %>%
      rowSums(na.rm = TRUE)) %>%
  mutate(Vasopressor=if_else(Vasopressor==0,0,1),
         NorepiEq=ifelse(NorepiEq>1,
                         sample(seq(0.9,1.1,0.01),sum(NorepiEq>1),replace =
T),
                         NorepiEq))
```
#eICU database reformat
```{r eICUdata}
library(data.table)
patient <- fread(input="zcat < /Users/zhang/Documents/eicu/patient.csv.gz",
                      sep = ',',header = T,
                 stringsAsFactors=T,
                 na.strings=c("",'Unknown','Other','NA'))
nurseCharting <- fread(input="zcat < /Users/zhang/Documents/eicu/
nurseCharting.csv.gz",
                       sep = ',',header = T)#to extract GCS score
lab<-fread(input="zcat < /Users/zhang/Documents/eicu/lab.csv.gz",
           sep = ',',header = T)
apachePatientResult <- fread(input="zcat < /Users/zhang/Documents/eicu/
apachePatientResult.csv.gz",
                       sep = ',',header = T)
maxICUday=8

lactate <- lab[labname=='lactate',]
lactate[,labresult:=as.numeric(labresult)]
lactate[,lactatedays:=cut(labresultoffset,breaks = seq(0,60*24*maxICUday,
60*24),
                  labels = seq(1,maxICUday))]
lactate <- lactate[!is.na(lactatedays),]
lactate <- lactate[,.(LAC.max=max(labresult,na.rm = T)),
           by = .(patientunitstayid, lactatedays)]
lactate <- lactate[is.finite(LAC.max),]

pH <- lab[labname=='pH',]
pH[,labresult:=as.numeric(labresult)]
pH[,pHdays:=cut(labresultoffset,breaks = seq(0,60*24*maxICUday,60*24),
                  labels = seq(1,maxICUday))]
pH <- pH[!is.na(pHdays),]
pH <- pH[,.(PH.min=min(labresult,na.rm = T)),
           by = .(patientunitstayid, pHdays)]
pH <- pH[is.finite(PH.min),]

cr<-lab[labname=='creatinine',]
cr[,labresult:=as.numeric(labresult)]
cr[,crdays:=cut(labresultoffset,breaks = seq(0,60*24*maxICUday,60*24),
                  labels = seq(1,maxICUday))]
````

```
cr <- cr[!is.na(crdays),]
cr <- cr[,.(Cre=max(labresult,na.rm = T)),
           by = .(patientunitstayid, crdays)]
cr <- cr[is.finite(Cre),]
platelet <- lab[labname=='platelets x 1000',]
platelet[,labresult:=as.numeric(labresult)]
platelet[,plateletdays:=cut(labresultoffset,breaks = seq(0,60*24*maxICUday,
60*24),
                              labels = seq(1,maxICUday))]
platelet <- platelet[!is.na(plateletdays),]
platelet <- platelet[,.(plateletval=min(labresult,na.rm = T)),
                       by = .(patientunitstayid, plateletdays)]
platelet <- platelet[is.finite(plateletval),]
pao2 <- lab[labname=='paO2',]
pao2[,labresult:=as.numeric(labresult)]
pao2[,pao2days:=cut(labresultoffset,breaks = seq(0,60*24*maxICUday,60*24),
                            labels = seq(1,maxICUday))]
pao2 <- pao2[!is.na(pao2days),]
pao2 <- pao2[,.(pao2val=min(labresult,na.rm = T)),
                     by = .(patientunitstayid, pao2days)]
pao2 <- pao2[is.finite(pao2val),]
fio2 <- lab[labname=='FiO2',]
fio2[,labresult:=as.numeric(labresult)]
fio2[,fio2days:=cut(labresultoffset,breaks = seq(0,60*24*maxICUday,60*24),
                    labels = seq(1,maxICUday))]
fio2 <- fio2[!is.na(fio2days)&labresult<=100,]
fio2 <- fio2[,.(fio2val=max(labresult,na.rm = T)),
             by = .(patientunitstayid, fio2days)]
fio2 <- fio2[is.finite(fio2val),]

paco2 <- lab[labname=='paCO2',]
paco2[,labresult:=as.numeric(labresult)]
paco2[,paco2days:=cut(labresultoffset,breaks = seq(0,60*24*maxICUday,
60*24),
                            labels = seq(1,maxICUday))]
paco2 <- paco2[!is.na(paco2days),]
paco2 <- paco2[,.(PCO2.max=max(labresult,na.rm = T)),
                     by = .(patientunitstayid, paco2days)]
paco2 <- paco2[is.finite(PCO2.max),]

HCO3 <- lab[labname=='HCO3',]
HCO3[,labresult:=as.numeric(labresult)]
HCO3[,HCO3days:=cut(labresultoffset,breaks = seq(0,60*24*maxICUday,60*24),
                            labels = seq(1,maxICUday))]
HCO3 <- HCO3[!is.na(HCO3days),]
HCO3 <- HCO3[,.(HCO3.min=min(labresult,na.rm = T)),
                     by = .(patientunitstayid, HCO3days)]


Hgb <- lab[labname=='Hgb',]
Hgb[,labresult:=as.numeric(labresult)]
Hgb[,Hgbdays:=cut(labresultoffset,breaks = seq(0,60*24*maxICUday,60*24),
                          labels = seq(1,maxICUday))]
```

```
Hgb <- Hgb[!is.na(Hgbdays),]
Hgb <- Hgb[,.(Hgbval=max(labresult,na.rm = T)),
                   by = .(patientunitstayid, Hgbdays)]
Hgb <- Hgb[is.finite(Hgbval),]
Hct <- lab[labname=='Hct',]
Hct[,labresult:=as.numeric(labresult)]
Hct[,Hctdays:=cut(labresultoffset,breaks = seq(0,60*24*maxICUday,60*24),
                          labels = seq(1,maxICUday))]
Hct <- Hct[!is.na(Hctdays),]
Hct <- Hct[,.(Hct=max(labresult,na.rm = T)),
                   by = .(patientunitstayid, Hctdays)]
Hct <- Hct[is.finite(Hct),]

RDWCV <- lab[labname=='RDW',]
RDWCV[,labresult:=as.numeric(labresult)]
RDWCV[,RDWCVdays:=cut(labresultoffset,breaks = seq(0,60*24*maxICUday,
60*24),
                          labels = seq(1,maxICUday))]
RDWCV <- RDWCV[!is.na(RDWCVdays),]
RDWCV <- RDWCV[,.(RDWCV=max(labresult,na.rm = T)),
                   by = .(patientunitstayid, RDWCVdays)]
RDWCV <- RDWCV[is.finite(RDWCV),]

BaseExcess <- lab[labname=='Base Excess',]
BaseExcess[,labresult:=as.numeric(labresult)]
BaseExcess[,BEdays:=cut(labresultoffset,breaks = seq(0,60*24*maxICUday,
60*24),
                          labels = seq(1,maxICUday))]
BaseExcess <- BaseExcess[!is.na(BEdays),]
BaseExcess <- BaseExcess[,.(BE.max=max(labresult,na.rm = T)),
                   by = .(patientunitstayid, BEdays)]
BaseExcess <- BaseExcess[is.finite(BE.max),]

Temper <- nurseCharting[grep('Temperature \\(F\\)|Temperature \\(C\\)',
                             nurseCharting$nursingchartcelltypevalname),]
Temper[,nursingchartvalue:=as.numeric(nursingchartvalue)]
Temper[,Temperdays:=cut(nursingchartoffset,breaks = seq(0,60*24*maxICUday,
60*24),
                labels = seq(1,maxICUday))]
Temper <- Temper[!is.na(Temperdays),];
#transform between celsus and F
Temper <- Temper[,nursingchartvalue :=
ifelse(nursingchartcelltypevalname=="Temperature (F)",
                                                 (nursingchartvalue-32)/1.8,
                                                 nursingchartvalue)]
Temper <- Temper[,.(Temper=max(nursingchartvalue,na.rm = T)),
         by = .(patientunitstayid, Temperdays)]
Temper <- Temper[is.finite(Temper),]
mBP <- nurseCharting[grep('BP
Systolic',nurseCharting$nursingchartcelltypevalname),]
mBP[,nursingchartvalue:=as.numeric(nursingchartvalue)]
mBP[,mBPdays:=cut(nursingchartoffset,breaks = seq(0,60*24*maxICUday,60*24),
                  labels = seq(1,maxICUday))]
```

```
mBP <- mBP[!is.na(mBPdays),];
mBP <- mBP[,.(mBP=min(nursingchartvalue,na.rm = T)),
           by = .(patientunitstayid, mBPdays)]
mBP <- mBP[is.finite(mBP),]

HR <- nurseCharting[grep('Heart
Rate',nurseCharting$nursingchartcelltypevalname),]
HR[,nursingchartvalue:=as.numeric(nursingchartvalue)]
HR[,HRdays:=cut(nursingchartoffset,breaks = seq(0,60*24*maxICUday,60*24),
                  labels = seq(1,maxICUday))]
HR <- HR[!is.na(HRdays),];
HR <- HR[,.(HR=max(nursingchartvalue,na.rm = T)),
           by = .(patientunitstayid, HRdays)]
HR <- HR[is.finite(HR),]
#preprocessing for infusion drugs
infusionDrug <- fread(input="zcat < /Users/zhang/Documents/eicu/
infusionDrug.csv.gz",
                      sep = ',',header = T)
infusionDrug[,drugrate:=as.numeric(drugrate)]
meanWeight <- infusionDrug[,mean(patientweight,na.rm = T)]
Dopamine <- infusionDrug[grep('Dopamine',infusionDrug$drugname),]
Dopamine <- Dopamine[,.
(patientunitstayid,infusionoffset,drugname,drugrate)]
Dopamine[,drugrateStand:=ifelse(drugname=='Dopamine (ml/hr)',#convert to
mcg/kg/min by assuming mean weight
                                drugrate*400*1000/
(250*60*meanWeight),drugrate)]
Dopamine[,infusiondays:=cut(infusionoffset,breaks = seq(0,60*24*maxICUday,
60*24),
                            labels = seq(1,maxICUday))]
Dopamine <- Dopamine[,.(dopaminerate=max(drugrateStand,na.rm = T)),
                     by = .(patientunitstayid, infusiondays)]
Dopamine <- Dopamine[is.finite(dopaminerate),]
#norepinephrine
Norepi <- infusionDrug[grep('Norepinephrine',infusionDrug$drugname),]
Norepi <- Norepi[,.(patientunitstayid,infusionoffset,drugname,drugrate)]
Norepi[,drugrateStand:=ifelse(grepl('(mcg/min)',drugname),#convert to mcg/
kg/min by assuming mean weight
                                drugrate/meanWeight,
                              ifelse(drugname=='Norepinephrine (ml/hr)',
                                     drugrate*4*1000/(250*60*meanWeight),
                                     ifelse(drugname=='Norepinephrine (mcg/
kg/hr)',
                                            drugrate/
meanWeight,drugrate)))]
Norepi[,infusiondays:=cut(infusionoffset,breaks = seq(0,60*24*maxICUday,
60*24),
                            labels = seq(1,maxICUday))]
Norepi <- Norepi[,.(Norepirate=max(drugrateStand,na.rm = T)),
                     by = .(patientunitstayid, infusiondays)]
Norepi <- Norepi[is.finite(Norepirate),]
Epinephrine <- infusionDrug[grep('Epinephrine',infusionDrug$drugname),]
```

```
Epinephrine <- Epinephrine[,.
(patientunitstayid,infusionoffset,drugname,drugrate)]
Epinephrine[,drugrateStand:=ifelse(drugname=='Epinephrine (ml/hr)',#convert
to mcg/kg/min by assuming mean weight
                                drugrate*4*1000/(250*60*meanWeight),
                                ifelse(drugname=='Epinephrine (mcg/min)',
                                       drugrate/meanWeight,drugrate))]
Epinephrine[,infusiondays:=cut(infusionoffset,breaks =
seq(0,60*24*maxICUday,60*24),
                            labels = seq(1,maxICUday))]
Epinephrine <- Epinephrine[,.(Epinephrinerate=max(drugrateStand,na.rm =
T)),
                     by = .(patientunitstayid, infusiondays)]
Epinephrine <- Epinephrine[is.finite(Epinephrinerate),]
##fluid balance
intakeOutput <- fread(input="zcat < /Users/zhang/Documents/eicu/
intakeOutput.csv.gz",
                      sep = ',',header = T)
intakeOutput[,intakeOutputdays:=cut(intakeoutputoffset,breaks =
seq(0,60*24*maxICUday,60*24),
                labels = seq(1,maxICUday))]
intakeOutput <- intakeOutput[!is.na(intakeOutputdays),]
#remove duplicated entry for intake
intakeOutput <- unique(intakeOutput,by =
c("patientunitstayid","intakeoutputoffset",
                                           "intaketotal","outputtotal"))
intakeOutput <- intakeOutput[,.(intaketotal = sum(intaketotal,na.rm = T),
                                outputtotal=sum(outputtotal,na.rm = T),
                                nettotal = sum(nettotal,na.rm = T),
                                urineOutput =
sum(outputtotal[grep("Urine",celllabel)],na.rm = T)),
         by = .(patientunitstayid, intakeOutputdays)]

#主表格
dtsepsis <- patient %>%
  filter(str_detect(apacheadmissiondx,"Sepsis")&hospitaldischargestatus!=''
) %>%
  dplyr::mutate(age=as.numeric(age)) %>%
  dplyr::select(patientunitstayid,gender,age,hospitaldischargestatus,admissionheight,
                unitdischargeoffset,hospitaldischargeoffset)
#将 ICU天数进行归一化
days <- dtsepsis[,.(patientunitstayid,unitdischargeoffset)]
days[,days:=ceiling(unitdischargeoffset/(60*24))]
days <- days[ ,.(days=1:days),by=c("patientunitstayid")]
days <- days[days<=maxICUday&days>=0,];
days[,days:=as.factor(days)]
dtEICU <- dtsepsis %>%
  right_join(
    days,by='patientunitstayid'
  ) %>%
  left_join(
    HR,
```

```
  by=c("patientunitstayid"="patientunitstayid","days"="HRdays")
) %>%
left_join(
  mBP,
  by=c("patientunitstayid"="patientunitstayid","days"="mBPdays")
) %>%
left_join(
  Temper,
  by=c("patientunitstayid"="patientunitstayid","days"="Temperdays")
) %>%
left_join(
  lactate,
  by=c("patientunitstayid"="patientunitstayid","days"="lactatedays")
)%>%
left_join(
  cr,
  by=c("patientunitstayid"="patientunitstayid","days"="crdays")
)%>%
left_join(
  platelet,
  by=c("patientunitstayid"="patientunitstayid","days"="plateletdays")
)%>%
left_join(
  pao2,
  by=c("patientunitstayid"="patientunitstayid","days"="pao2days")
)%>%
left_join(
  fio2,
  by=c("patientunitstayid"="patientunitstayid","days"="fio2days")
)%>%
left_join(
  paco2,
  by=c("patientunitstayid"="patientunitstayid","days"="paco2days")
)%>%
left_join(
  HCO3,
  by=c("patientunitstayid"="patientunitstayid","days"="HCO3days")
)%>%
left_join(
  Hgb,
  by=c("patientunitstayid"="patientunitstayid","days"="Hgbdays")
)%>%
left_join(
  Hct,
  by=c("patientunitstayid"="patientunitstayid","days"="Hctdays")
)%>%
left_join(
  RDWCV,
  by=c("patientunitstayid"="patientunitstayid","days"="RDWCVdays")
)%>%
left_join(
  BaseExcess,
  by=c("patientunitstayid"="patientunitstayid","days"="BEdays")
```

```
  )%>%
  left_join(
    Dopamine,
    by=c("patientunitstayid"="patientunitstayid","days"="infusiondays")
  )%>%
  left_join(
    Norepi,
    by=c("patientunitstayid"="patientunitstayid","days"="infusiondays")
  )%>%
  left_join(
    Epinephrine,
    by=c("patientunitstayid"="patientunitstayid","days"="infusiondays")
  )%>%
  left_join(
    intakeOutput,
    by=c("patientunitstayid"="patientunitstayid","days"="intakeOutputdays")
  )%>%
  left_join(
    apachePatientResult %>%
      distinct(patientunitstayid,.keep_all=T) %>%
      dplyr::select(patientunitstayid,apachescore),
    by=c("patientunitstayid"="patientunitstayid")
  ) %>%
  left_join(
    pH,
    by=c("patientunitstayid"="patientunitstayid","days"="pHdays")
  ) %>%
  dplyr::mutate(
    fio2val=if_else(fio2val<=1,fio2val*100,
                    if_else(fio2val<21,21,fio2val))
  ) %>%
  dplyr::mutate(
    APACHEII = apachescore*71/299,
    Weight=if_else(gender=="Female",45.5 + 2.3*(admissionheight/2.54 - 60),
                   50 + 2.3 *(admissionheight/2.54 - 60) ),
    PF.min = pao2val*100/fio2val
    ) %>%
  dplyr::rename(
    "Age"="age","HR.max"="HR","BP.min"="mBP",
    "Temp.max"="Temper","Plt"="plateletval",
    "Urine"="urineOutput", "OutputVol"= "outputtotal",
    "IntakeVol" = "intaketotal",
    "Gender"="gender"
  ) %>%
  dplyr::mutate(
      HR.max = if_else(HR.max>200,mean(HR.max,na.rm = T),HR.max),
    APACHEII = if_else(APACHEII<=0,mean(APACHEII,na.rm = T),APACHEII),
    BP.min = if_else(BP.min<10,median(BP.min,na.rm = T),BP.min),
    Temp.max =if_else(Temp.max>42,median(Temp.max,na.rm = T),Temp.max),
    PH.min = if_else(PH.min<5,median(PH.min,na.rm = T),PH.min),
    HCO3.min = if_else(HCO3.min<2,median(HCO3.min,na.rm = T),HCO3.min)
  )
#Identify patients with vasopressor on day 0 for septic shock
```

```
dtSepticshock <- dtEICU %>%
  dplyr::mutate(
    IndNoShock =
is.na(dopaminerate)&is.na(Norepirate)&is.na(Epinephrinerate)
  ) %>%
  group_by(patientunitstayid) %>%
  group_modify(
    ~{
      .x$day1_flgNO <-
        .x %>% filter(days=="1") %>%
          pull(IndNoShock)
      return(.x)
    }
  ) %>% ungroup() %>%
  filter(!day1_flgNO) %>%
    mutate(across(.cols = dopaminerate:Epinephrinerate,~{
    if_else(is.na(.x),0,.x)
  })) %>%
  group_by(patientunitstayid) %>%
  fill(everything(),.direction = "updown") %>% ungroup() %>%
    dplyr::mutate(across(.cols =
c(Age,Weight,HR.max,APACHEII,HR.max,BP.min,Temp.max,PH.min,
         HCO3.min,LAC.max,BE.max,PF.min,PCO2.max,Hct,Plt,RDWCV,Cre,Urine),
                       ~{
                         .x <- if_else(is.infinite(.x)|is.na(.x),
                                mean(.x[!is.infinite(.x)&!is.na(.x)]),
                                .x)
                return(.x)
                       }
                )
                ) %>%
  dplyr::mutate(days=as.numeric(days)-2,
                NorepiEq=rowSums(
                  select(.,
                         .dots = c("dopaminerate",
"Norepirate","Epinephrinerate")),
                  na.rm = T),
                mort=as.numeric(hospitaldischargestatus)-2 ,
                Hospital_LOS = hospitaldischargeoffset/(24*60),
                ICU_LOS = unitdischargeoffset/(24*60),
                Cre = Cre*88.4
           ) %>%
  dplyr::filter(days%in%c(0,1,2,3,7))
#description of the cohort
tab1_eICU <- twogrps(dtSepticshock[dtSepticshock$days==0,],
                    gvar = "mort",
                    varlist = c("Age","Gender","Weight",
              "APACHEII","dopaminerate",
              "Norepirate","Epinephrinerate",
              "HR.max","BP.min","Temp.max",
              "HCO3.min","BE.max","LAC.max",
              "PF.min","PCO2.max","Hgbval","Hct",
              "Plt","RDWCV","Cre",
```

```
              "IntakeVol","OutputVol","Urine",
              "Hospital_LOS","ICU_LOS")
           )
```

```
#Latent profile analysis for multicohort DataSet
```{r LPA}
library(tidyLPA)
dtClustering <- dtfinal%>%
  select(Age,Weight,HR.max,APACHEII,HR.max,BP.min,Temp.max,PH.min,
         HCO3.min,LAC.max,BE.max,PF.min,PCO2.max,Hct,Plt,RDWCV,Cre,Urine)
set.seed(4)
modLPA <- dtClustering %>%
    estimate_profiles(2:10,verbose=T)
#https://cran.r-project.org/web/packages/tidyLPA/vignettes/
Introduction_to_tidyLPA.html
PLAcriteria <- modLPA %>% get_fit() %>%
  mutate(across(.cols = everything(),~round(.x,3))) %>%
  select(Classes,AIC,SABIC,Entropy,prob_min,prob_max,n_min,n_max,BLRT_p)
%>%
  mutate(AIC=round(AIC),SABIC=round(SABIC)) %>%
  ggtexttable(rows = NULL, theme = ttheme("mOrange")) %>%
  tab_add_title(text = "Best Number of Classes for FMM",
                face = "bold", padding = unit(1.5, "line"),
                just=c(-0.8,1))
set.seed(4)
LPA_5class <- dtClustering%>%
  estimate_profiles(n_profiles=5)
dtClass <- dtfinal %>%
  mutate(id=rep(1:(length(name)/5),each=5)) %>%
  bind_cols(Class=get_data(LPA_5class)$Class) %>%
  mutate(Class=if_else(Class==1,2,
                       if_else(Class==2,1,Class))) %>%
  filter(days<=Hospital_LOS) %>%
  group_by(Hospital,name) %>%
  group_modify(~{
    .x$Nextclass <- c(.x$Class[-1],if_else(.x$Mort[1]==1,7,6))
    return(.x)
  }) %>% ungroup() %>%
  mutate(Reward=if_else(Nextclass==6,1,
                        if_else(Nextclass==7,-1,0)),
         Action=as.character(cut(IntakeVol,breaks =
c(50,2500,3500,4000,100000),
                              labels = c("1","2","3","4"))),
         Class=as.character(Class),
         Nextclass=as.character(Nextclass),
         Mort = factor(Mort,levels = c(0,1),labels =c("Alive","Dead"))
         ) %>% as.data.frame()
#Alluvial Plots
plotAlluvia <- dtClass %>%
  select(Class,days,id,Mort) %>%
  mutate(days=as_factor(days)
         ) %>%
```
```

~~~
  ggplot(
  aes(x = days, stratum = Class, alluvium = id,
           label = Class,fill=Class)) +
  geom_flow(aes(color = Class),stat = "alluvium",
            lode.guidance = "frontback",
            ) +
  scale_fill_lancet()+
  scale_color_lancet(alpha=0.3)+
  geom_stratum() +
  theme(legend.position = "bottom") +
  labs(title="Sepsis State Transition Over Time",
       x="Days after Hospital Admission",
       ylab="Number of Patients")+
  facet_wrap(~ Mort, scales = "free_y")+theme_bw()
#Characteristics of each Class type
laby <- dtClass %>%
  mutate(Class=factor(Class,levels = 1:5,
                      labels = paste("Class",1:5))) %>%
  mutate(across(.cols = c(Age,Weight,APACHEII,HR.max:Cre,IntakeVol:Urine),
                scale)) %>%
  pivot_longer(cols = c(Age,Weight,APACHEII,HR.max:Cre,IntakeVol:Urine),
               names_to = "Items",
               values_to = "Scaled value") %>%
  group_by(Items,Class) %>%
  summarise(Mean=mean(`Scaled value`)) %>%
  group_by(Items)%>%
  summarise(Max=max(Mean))
laby <- laby[match(dtClass %>%
                   select(c(Age,Weight,APACHEII,
                            HR.max:Cre,IntakeVol:Urine)) %>%
                   colnames(),
                 laby$Items),] %>% pull(Max)
plot_Class <- dtClass %>%
  mutate(Class=factor(Class,levels = 1:5,
                      labels = paste("Class",1:5))) %>%
  mutate(across(.cols = c(Age,Weight,APACHEII,HR.max:Cre,IntakeVol:Urine),
                scale)) %>%
  pivot_longer(cols = c(Age,Weight,APACHEII,HR.max:Cre,IntakeVol:Urine),
               names_to = "Items",
               values_to = "Scaled value") %>%
  ggline(x = "Items", y = "Scaled value", add = "mean_ci",
          color = "Class", palette = "lancet",
         position=position_dodge(width = 0.2),
         error.plot="errorbar")+
  stat_compare_means(aes(group = Class), label = "p.signif",
                     label.y = laby)+
  theme_pubr(x.text.angle=45)+
  labs(x="")
```

#Kmeans Clustering to confirm the number of clusters
```{r KmeansClustering}
corplot <- dtfinal%>%
~~~

```
  select(where(is.numeric)) %>%
  select(-c(平衡量,小时数_0)) %>%
  DataExplorer::plot_correlation(theme_config=list(legend.position="right",
                                                  axis.text.x =
element_text(angle = 45)))


library(NbClust)
nbclust_out <- dtClustering %>%
  select(-Urine) %>%
  NbClust(
  distance = "euclidean",
  min.nc = 2, # minimum number of clusters
  max.nc = 10, # maximum number of clusters
  method = "kmeans" # one of: "ward.D", "ward.D2", "single", "complete",
"average", "mcquitty", "median", "centroid", "kmeans"
)

# create criteria plot
plot_kmeanCriteria <- nbclust_out$All.index %>%
  as.data.frame() %>%
  mutate(Ncluster=2:10) %>%
  mutate(across(-Ncluster,scale)) %>%
  pivot_longer(cols = -Ncluster,
               names_to="Index",
               values_to="Scaled Value") %>%
ggplot(aes(x = Ncluster,y=`Scaled Value`,color=Index)) +
  geom_line () +geom_point(aes(shape=Index))+
  scale_shape_manual(values=seq(0,10))+
  scale_color_lancet()+
  labs(x = "Number of clusters", y = "Scaled Value", title = "Optimal
number of clusters by K-means") +
  theme_minimal()
km_res <- dtfinal%>%
  select(Age,Weight,HR.max,APACHEII,HR.max,BP.min,Temp.max,PH.min,
         HCO3.min,LAC.max,PF.min,PCO2.max,Hct,Plt,RDWCV,Cre) %>%
  kmeans(centers = 5, nstart = 20)
fviz_cluster(km_res,
             dtfinal%>%
  select(Age,Weight,HR.max,APACHEII,HR.max,BP.min,Temp.max,PH.min,
         HCO3.min,LAC.max,PF.min,PCO2.max,Hct,Plt,RDWCV,Cre),
  ellipse.type = "norm", stand=T,geom = "points"
  )
#Principal component analysis visualization
library(rayshader)
res.pca <- prcomp(dtClustering,  scale = TRUE)
PCA1 <- fviz_pca_ind(res.pca,axes = c(1, 2),geom = c("point"),
             col.ind = as.factor(if_else(get_data(LPA_5class)$Class==1,2,
                                 if_else(get_data(LPA_5class)
$Class==2,1,
                                         get_data(LPA_5class)
$Class))),
             addEllipses =T)+
```

```
  scale_color_lancet()
PCA2 <- fviz_pca_ind(res.pca,axes = c(1, 3),geom = c("point"),
             col.ind = as.factor(if_else(get_data(LPA_5class)$Class==1,2,
                                         if_else(get_data(LPA_5class)
$Class==2,1,
                                                 get_data(LPA_5class)
$Class))),
             addEllipses =T)+
  scale_color_lancet()
#Combine all plots together
ggdraw() +
  draw_plot(plot_kmeanCriteria, x=0,y=0.7, width =  0.5, height =  0.3) +
  draw_plot(PLAcriteria, 0.5,0.7,  0.5, 0.3)+
  draw_plot(plotAlluvia, 0,0.3,  0.5, 0.4)+
  draw_plot(PCA1, 0.5,0.3,  0.5, 0.2)+
  draw_plot(PCA2, 0.5,0.5,  0.5, 0.2)+
  draw_plot(plot_Class, 0, 0, 1, 0.3) +
  draw_plot_label(c("A", "B","C","D","E"),
                  x=c(0,0.5, 0,0.5,0), y=c(1,1, 0.7,0.7,0.3), size = 15)
```

Interaction with fluid and vasopressor in logistic regression model

```{r InteractionMultiply}
library(visreg)
library(survival)
library("survminer")
library(ggeffects)
library(Publish)
dtLong <- dtfinal %>%
  bind_cols(Class=get_data(LPA_5class)$Class) %>%
  mutate(Class=if_else(Class==1,2,#baseline class is class 1
                     if_else(Class==2,1,Class))) %>%
  mutate(id=rep(1:(length(name)/5),each=5),
         Class=as_factor(Class),
         `小时数_0`=ifelse(`小时数_0`>24,24,`小时数_0`),
         IntakeVol = scale(IntakeVol,scale = 1000,center = F)
         ) %>%
  group_by(id) %>%
  group_modify(~{
    .x$daysEnd <- c(.x$days[-1],.x$Hospital_LOS[1])
    .x <- .x[.x$days<.x$daysEnd,]
    .x$Mortality <- c(rep(0,nrow(.x)-1),.x$Mort[1])
    return(.x)
  })

modFluid <- coxph(Surv(days,daysEnd,Mortality)~Class*IntakeVol+
                    Comorbid+
                    APACHEII+Urine+Cre+Age,
            id=id,
           data = dtLong)
plot_Interaction <-visreg(modFluid,xvar = "IntakeVol",by="Class",trans =
exp,
       gg=T)+theme_bw()+facet_wrap(~Class,nrow = 1)+
```

```
   coord_cartesian(ylim = c(0, 10))+
  labs(x="Daily Fluid Intake (L)",
       y="Adjusted Hazard ratio")
plotTable <- ShowRegTable(modFluid) %>% as.data.frame() %>%
  rename("HR [95% CI]"="exp(coef) [confint]",
         "p value"="p") %>%
  rownames_to_column("Variable") %>%
  add_row(Variable = "Class 1 (as reference)",
          `HR [95% CI]` = "1",
          `p value` = "", .before = 1) %>%
  mutate(Variable=str_replace(Variable,
                              "^IntakeVol","Intake Vol (for every 1-L)"))
%>%
  mutate(Variable=str_replace(Variable,"Comorbid","")) %>%
  add_row(Variable = "Comorbidity (None as reference)",
          `HR [95% CI]` = "1",
          `p value` = "", .before = 7) %>%
  column_to_rownames("Variable") %>%
  ggtexttable() %>%
  table_cell_bg(row = c(7,17), column = 3,
                      linewidth = 3,color = "red")

modVaso <- coxph(Surv(days,daysEnd,Mortality)~Class*NorepiEq+
                   Comorbid+Age+IntakeVol+
                    APACHEII+Urine+Cre,
            id=id,
           data = dtLong)
plot_Interaction1 <-visreg(modVaso,xvar = "NorepiEq",by="Class",trans =
exp,
       gg=T)+theme_bw()+facet_wrap(~Class,nrow = 1)+
   coord_cartesian(ylim = c(0, 10))+
  labs(x="Daily Maximum dose of Norepinephrine (mcg/kg/min)",
       y="Adjusted Hazard ratio")
plotTable1 <- ShowRegTable(modVaso) %>% as.data.frame() %>%
  rename("HR [95% CI]"="exp(coef) [confint]",
         "p value"="p") %>%
  rownames_to_column("Variable") %>%
  add_row(Variable = "Class 1 (as reference)",
          `HR [95% CI]` = "1",
          `p value` = "", .before = 1) %>%
  mutate(Variable=str_replace(Variable,
                              "^IntakeVol","Intake Vol (for every 1-L)"))
%>%
  mutate(Variable=str_replace(Variable,"Comorbid","")) %>%
  add_row(Variable = "Comorbidity (None as reference)",
          `HR [95% CI]` = "1",
          `p value` = "", .before = 7) %>%
  column_to_rownames("Variable") %>%
  ggtexttable() %>%
  table_cell_bg(row = c(7,19), column = 3,
                      linewidth = 3,color = "red")
ggdraw() +
  draw_plot(plot_Interaction, x=0,y=0.8, width =  1, height =  0.2) +
```

~~~~
  draw_plot(plot_Interaction1, 0,0.6,  1, 0.2)+
  draw_plot(plotTable, 0,0,  0.5, 0.6)+
  draw_plot(plotTable1, 0.5,0,  0.5, 0.6)+
  draw_plot_label(c("A", "B","C","D"),
                  x=c(0,0, 0,0.5), y=c(1,0.8, 0.6,0.6), size = 15)
```


# DTR model
```{r DTR model}
library(DTRreg)
dtWide <- dtfinal %>%
  bind_cols(Class=get_data(LPA_5class)$Class) %>%
  mutate(Class=if_else(Class==1,2,
                       if_else(Class==2,1,Class))) %>%
  mutate(id=rep(1:(length(name)/5),each=5),
         Class=as_factor(Class),
         `小时数_0`=ifelse(`小时数_0`>24,24,`小时数_0`)
         ) %>%
  pivot_wider(id_cols = c(id,Age:Mort,`小时数_0`),
              names_from = days,
              values_from = c(Vasopressor,Norepi.max:Cre,
                              IntakeVol:Urine,Class)
              ) %>%
  mutate(#IntakeVol_0=IntakeVol_0*24/`小时数_0`,
         Y= (1-2*Mort)/(1+ICU_LOS+ 0.5*(Hospital_LOS-ICU_LOS))
         )
#Table 1 showing the baseline characteristics across classes
tab1 <- multigrps(
  dtWide,
  gvar = "Class_0",
  varlist = c("Age","Gender","Weight","Comorbid","SiteInfect",
              "小时数_0",
              "Type","MV","APACHEII",
              "Norepi.max_0",
              "HR.max_0","BP.max_0","BP.min_0","Temp.max_0","PH.min_0",
              "HCO3.min_0","BE.min_0","LAC.max_0",
              "PF.min_0","PCO2.max_0","Hb_0","Hct_0",
              "Plt_0","RDWCV_0","Cre_0",
              "IntakeVol_0","OutputVol_0","Urine_0",
              "Hospital_LOS","ICU_LOS","Mort"),
  sim=TRUE
  )

Xpsi1<-list(
  ~HR.max_0+BP.min_0+Temp.max_0+Class_0+Urine_0+Hct_0+
    PF.min_0+PH.min_0+LAC.max_0,
  ~HR.max_1+BP.min_1+Temp.max_1+Class_1+Urine_1+Hct_1+
    PF.min_1+PH.min_1+LAC.max_1,
  ~HR.max_2+BP.min_2+Temp.max_2+Class_2+Urine_2+Hct_2+
    PF.min_2+PH.min_2+LAC.max_2,
  ~HR.max_3+BP.min_3+Temp.max_3+Class_3+Urine_3+Hct_3+
    PF.min_3+PH.min_3+LAC.max_3,
~~~~

```
  ~HR.max_7+BP.min_7+Temp.max_7+Class_7+Urine_7+Hct_7+
    PF.min_7+PH.min_7+LAC.max_7
)
Xpsi2<-list(~Class_0,
            ~Class_1,
            ~Class_2,
            ~Class_3,
            ~Class_7)
treat.mod<-list(IntakeVol_0~Weight+HR.max_0+BP.min_0+Temp.max_0,
                IntakeVol_1~Weight+HR.max_1+BP.min_1+Temp.max_1,
                IntakeVol_2~Weight+HR.max_2+BP.min_2+Temp.max_2,
                IntakeVol_3~Weight+HR.max_3+BP.min_3+Temp.max_3,
                IntakeVol_7~Weight+HR.max_7+BP.min_7+Temp.max_7
                )
tf.mod<-list(~APACHEII+Age+Gender+RDWCV_0+Cre_0+Hct_0+LAC.max_0,
             ~ Age+Gender+RDWCV_1+Cre_1+Hct_1+LAC.max_1,
             ~ Age+Gender+RDWCV_2+Cre_2+Hct_2+LAC.max_2,
             ~ Age+Gender+RDWCV_3+Cre_3+Hct_3+LAC.max_3,
             ~ Age+Gender+RDWCV_7+Cre_7+Hct_7+LAC.max_7)
out1 <- gdwols(outcome = Y, Xpsi1=Xpsi1, Xpsi2 =Xpsi1, treat.mod,
               treat.fam = gaussian(link = "log"),
               tf.mod, weight.fcn="qpom",
               data=dtWide, m=20, k=5,
               treat.range=c(2000,5000))
optDT <- do.call(cbind.data.frame, out1$opt.treat) %>%
  bind_cols(dtWide) %>%
  rename_with(.cols = contains("c("),
              ~ str_replace(.x,".*",paste("OptFluid",c(0,1,2,3,7),sep =
"_"))  ) %>%
  pivot_longer(cols = ends_with(c("_0","_1","_2","_3","_7")),
               names_to = c(".value", "Days"),
               names_sep= -2) %>%
  mutate(Days=parse_number(Days)) %>%
  mutate(deltaIntakeVolcont=IntakeVol-OptFluid,
    IntakeVolcat=cut(deltaIntakeVolcont,
              breaks = c(-6000,-2000,-500,500,1500,200000),
              labels = c("Very Low","Low","Optimal","High","Very High")
              )) %>%
  mutate(IntakeVolcat = fct_relevel(IntakeVolcat,"Optimal"))
#Impact of difference between actual versus Optimal on Mortality Outcome
mod_MortDelta <- glm(Mort~deltaIntakeVolcont+I(deltaIntakeVolcont^2)
+APACHEII+Age,
                     data = optDT,
                     family = "binomial")
plot_MortDelta <- visreg::visreg(mod_MortDelta,
                                 xvar ="deltaIntakeVolcont",
                                 scale = "response",gg=T)+
 # ylim(0.25,0.33)+
  labs(x="Difference between Actual and Optimal MP (Joules/min)",
       y="Probability of Hospital Death")
mod_MortDelta <- glm(Mort~IntakeVolcat,data = optDT,family = "binomial")
plot_MortDelta_tab <- publish(mod_MortDelta)$regressionTable %>%
  as.data.frame()   %>%
```

````
  rename("Odds Ratio"="OddsRatio",
         "95% CI"="CI.95") %>%
  select(-Variable) %>%
  column_to_rownames("Units") %>%
  ggtexttable() %>%
  tab_add_title(text = "Impact of Delta MP on Mortality",
                face = "bold", padding = unit(1.5, "line"))
plot_IntakeVol_Optimal <- optDT %>%
  mutate(Class=paste("Class",Class),
         Days=as_factor(Days)
         ) %>%
  rename(`Fluid Intake Category`=IntakeVolcat) %>%
  ggplot(aes(x=Days,y=Age,fill=`Fluid Intake Category`))+
  geom_bar(position = "fill",stat = "identity") +
  facet_wrap(~Class,scales="free_y",ncol=5)+
  scale_fill_lancet()+theme(legend.position="top")+
  labs(y="Number of Patients",
       x="Days after ICU entry")

panels <- default_forest_panels(
    model = ModRF,
    factor_separate_line = FALSE,
    measure = NULL,
    trans_char = "I"
)
panels <- panels[-4]
Plot_forest <- forest_model(ModRF,
             format_options =forest_model_format_options(
               text_size = 5,point_size = 5,colour = "red"
               ),panels)+
  labs(title = "Risk Factors for Hyperventilation")+
  theme(plot.title = element_text(hjust = 0.5))

ggdraw() +
  draw_plot(plot_MPstat_Disease, 0,0.7, 0.65, 0.3)+
  draw_plot(plot_MPstat_OptProp, x=0,y=0.4, width =  0.65, height =  0.3) +
  draw_plot(plot_MortDelta_tab, 0.65,0.7,  .35, 0.3)+
  draw_plot(plot_MortDelta, 0.65,0.4,  .35, 0.3)+
  draw_plot(Plot_forest, 0,0,  1, 0.4)+
  draw_plot_label(c("A", "B","C","D","E"),
                  x=c(0,0, 0.65, 0.65,0), y=c(1,0.7,1,0.7, 0.4), size = 15)
```

#risk factors for fluid overload
```{r RiskFactorFluidOverload}
library(rms)
dtFluidOver <- optDT %>%
  pivot_longer(cols = matches("_[01237]"),
               names_to=c(".value", "Days"),
               names_sep="_"
               ) %>%
  mutate(#Days=as.factor(Days),
         FluidOver = IntakeVol-OptFluid > 1000,
````

````
         FluidUnder = OptFluid - IntakeVol > 1000) %>%
  mutate(Plt=scale(Plt,scale = 20)[,1],
         HR.max = scale(HR.max,scale = 20)[,1],
         Cre=scale(Cre,scale = 20)[,1],
         BE.max=scale(BE.max,scale=5)[,1],
         BE.min=scale(BE.min,scale=5)[,1],
         APACHEII=scale(APACHEII,scale = 10)[,1],
         Age=scale(Age,scale = 10)[,1]) %>%
  mutate(Days=as.numeric(Days))
NamesFull <- dtFluidOver %>%
  dplyr::select(Age:Weight,Comorbid:APACHEII,HR.max:Cre,Urine) %>%
  colnames()
modstepwise <- lrm(as.formula(
  paste("FluidOver",
        paste(c("Class*Days",NamesFull),collapse = "+"),sep = "~")),
               data = dtRiskFactor,x=T,y=T) %>%
  fastbw(rule="p",sls=0.01)
as.formula(paste("FluidOver",
        paste(modstepwise$names.kept,collapse = "+"),sep = "~"))
modOver <- glm(
  FluidOver ~ Age + SiteInfect + Type + MV + APACHEII +
    HR.max + Norepi + PH.min + Hct + Plt + Cre + Class * Days,
  data = dtFluidOver
)
plot_fluidOver <- visreg(modOver,
                        xvar = "Days",by="Class",gg=T,scale = "response")+
  theme_bw()+facet_wrap(~Class,nrow = 1)+
   coord_cartesian(ylim = c(0, 0.7))+
  labs(x="",
       y="Probablity of Fluid Overloading")
plot_TabFluidOver <- ShowRegTable(modOver)%>%
  as.data.frame() %>%
  slice(-1) %>%
  rename("OR [95% CI]"="exp(coef) [confint]") %>%
  ggtexttable()
  table_cell_bg(row = c(6), column = 3,
                      linewidth = 3,color = "red")
```

#risk factor explored by using nneural network algorithm
```{r RiskFctNeuralNetwork}
library("SHAPforxgboost")
library(xgboost)
xSet <- dtFluidOver %>%
  dplyr::select(Age:Weight,Comorbid:APACHEII,Days,HR.max:Cre,Urine,Class)
xSet <- model.matrix(~.,xSet)
ySet <- dtFluidOver %>%
  dplyr::mutate(FluidOver=as.numeric(FluidOver)) %>%
  pull(FluidOver)
param_list <- list(objective = "binary:logistic",  # For regression
                   eta = 0.02,
                   max_depth = 5,
                   gamma = 0.01,
````

````
                    subsample = 0.95
                    )
modFluidRFxgboost <- xgboost::xgboost(data = xSet,
                                         label = ySet,
                         params = param_list, nrounds = 10,
                         verbose = FALSE,
                         early_stopping_rounds = 8)
# To return the SHAP values and ranked features by mean|SHAP|
shap_values <- shap.values(xgb_model = modFluidRFxgboost, X_train = xSet)
# option 2: supply a self-made SHAP values dataset (e.g. sometimes as
output from cross-validation)
SHARP_fluid <- shap.plot.summary.wrap1(model = modFluidRFxgboost,  X =
xSet,top_n=20)
depSHARP_fluid <- shap.plot.dependence(
  data_long = shap.prep(
    xgb_model = modFluidRFxgboost,  X_train = xSet,top_n=20
    ),
  x = 'Days',
  y = 'Days', color_feature = 'Days') +
  ggtitle("SHAP values of Days vs. Days")
```

# fluid type and Vasopressor with DTR
```{r FluidTypeDTR}
Xpsi1 = list(
    ~HR.max_0+BP.min_0+Temp.max_0+Class_0+Urine_0+Hct_0+
    PF.min_0+PH.min_0+LAC.max_0,
    ~HR.max_1+BP.min_1+Temp.max_1+Class_1+Urine_1+Hct_1+
    PF.min_1+PH.min_1+LAC.max_1,
    ~HR.max_2+BP.min_2+Temp.max_2+Class_2+Urine_2+Hct_2+
    PF.min_2+PH.min_2+LAC.max_2,
  ~HR.max_3+BP.min_3+Temp.max_3+Class_3+Urine_3+Hct_3+
    PF.min_3+PH.min_3+LAC.max_3,
  ~HR.max_7+BP.min_7+Temp.max_7+Class_7+Urine_7+Hct_7+
    PF.min_7+PH.min_7+LAC.max_7
  )
DTRmodVaso <- gdwols(
  outcome = Y,
  Xpsi1 = Xpsi1,
  Xpsi2 = Xpsi1,
  treat.mod = list(
    Norepi.max_0~Weight+HR.max_0+BP.min_0+Temp.max_0,
    Norepi.max_1~Weight+HR.max_1+BP.min_1+Temp.max_1,
    Norepi.max_2~Weight+HR.max_2+BP.min_2+Temp.max_2,
    Norepi.max_3~Weight+HR.max_3+BP.min_3+Temp.max_3,
    Norepi.max_7~Weight+HR.max_7+BP.min_7+Temp.max_7),
  tf.mod = list(~APACHEII+Age+Gender+RDWCV_0+Cre_0+Hct_0+LAC.max_0,
             ~ Age+Gender+RDWCV_1+Cre_1+Hct_1+LAC.max_1,
             ~ Age+Gender+RDWCV_2+Cre_2+Hct_2+LAC.max_2,
             ~ Age+Gender+RDWCV_3+Cre_3+Hct_3+LAC.max_3,
             ~ Age+Gender+RDWCV_7+Cre_7+Hct_7+LAC.max_7),
  weight.fcn="qpom",
               data=dtWide, m=10, k=5,
````

```r
  treat.range=c(0,1)
  )
dtNorepi <- do.call(
  cbind.data.frame,
  DTRmodVaso$opt.treat
  ) %>%
  bind_cols(dtWide) %>%
  rename_with(
    .cols = contains("c("),
    ~ str_replace(.x,".*",paste("OptVaso",
                                c(0,1,2,3,7),sep = "_"))
    ) %>%
  pivot_longer(cols = c(Vasopressor_0:Class_7,
                       OptVaso_0:OptVaso_7
                       ),
               names_to = c(".value", "Days"),
               names_sep="_") %>%
  mutate(Epinephrineflg = Norepi.max-OptVaso >0.2 ,
         Urine=scale(Urine,scale = 500)[,1],
         RDWCV=scale(RDWCV,scale = 5)[,1],
         HR.max=scale(HR.max,scale = 20)[,1],
         HCO3.max=scale( HCO3.max,scale = 10)[,1],
         Hb=scale(Hb,scale = 20)[,1],
         APACHEII=scale(APACHEII,scale = 10)[,1]) %>%
  mutate(Days=as.numeric(Days))
plot_vaso <- dtNorepi %>%
  dplyr::select(Days,Norepi.max,OptVaso,id,Class) %>%
  pivot_longer(cols = c(Norepi.max,OptVaso),
              names_to="Epinephrine",
              values_to="Dose") %>%
  mutate(Epinephrine=if_else(
    Epinephrine=="Norepi.max","Actual","Optimal")) %>%
  ggline(x="Days",y="Dose",color="Epinephrine",
         palette = "lancet",
         add = "mean_ci",facet.by="Class",scales = "free_y",
         panel.labs = list(Class = paste("Class",1:5)),
         position=position_dodge(width = 0.2),nrow=1,xlab = "")+
  theme(legend.position="top") +
  stat_compare_means(aes(group = `Epinephrine`),
                     label = "p.signif",
                     label.y = 1.3)+
  coord_cartesian(ylim=c(0,1.5))+
  labs(y="Dose (mcg/kg/min)")
#risk factor for inappropriately using Epinephrine
NamesFull <- dtNorepi %>%
  dplyr::select(Age:Weight,Comorbid:APACHEII,HR.max:Temp.min,PH.max:Cre,Urine)
%>%
  colnames()
modstepwise <- lrm(as.formula(
  paste("Epinephrineflg",
       paste(c("Class*Days",NamesFull),collapse = "+"),sep = "~")
  ),
               data = dtNorepi,x=T,y=T) %>%
```

```
  fastbw(rule="p",sls=0.05)
as.formula(paste("Epinephrineflg",
        paste(modstepwise$names.kept,collapse = "+"),sep = "~"))
modNorepiOver <- glm(
  Epinephrineflg ~ MV + APACHEII + HR.max + HR.min +
    PH.min + LAC.max + Hb + Hct + Plt + RDWCV + Urine +
    Class*Days,
  data = dtNorepi,family = "binomial"
)
plot_vasoOver <- visreg(modNorepiOver,
                        xvar = "Days",by="Class",gg=T,scale = "response")+
  theme_bw()+facet_wrap(~Class,nrow = 1)+
   coord_cartesian(ylim = c(0, 0.5))+
  labs(x="Days after ICU Admission",
       y="Probablity of Norepinephrine Over dose")
plot_TabvasoOver <- ShowRegTable(modNorepiOver)%>%
  as.data.frame() %>%
  slice(-1) %>%
  rename("OR [95% CI]"="exp(coef) [confint]") %>%
  ggtexttable()
  table_cell_bg(row = c(6), column = 3,
                      linewidth = 3,color = "red")
#combine all figures together
plot_Opt <- ggdraw() +
  draw_plot(plot_optfluid, x=0,y=.85, width =  1, height =  .15) +
  draw_plot(plot_vaso, x=0,y=0.7, width =1, height =  .15)+
  draw_plot(plot_fluidOver, x=0,y=0.55, width =1, height =  .15)+
  draw_plot(plot_vasoOver, x=0,y=0.4, width =1, height =  .15)+
  draw_plot(plot_TabFluidOver, x=0,y=0, width =0.5, height =  .4)+
  draw_plot(plot_TabvasoOver, x=0.5,y=0, width =0.5, height =  .4)+
  draw_plot_label(c("A", "B","C","D","E","F"),
                  x=c(0,0, 0,0,0,.5), y=c(1,.85, 0.7,0.57,0.4,.4), size =
15)
```

#Epinephrine risk factor

```{r RFnorepinephrineXGboost}
xSet_epi <-  dtNorepi%>%
  dplyr::select(Age:Weight,Comorbid:APACHEII,Days,HR.max:Temp.min,
              PH.max:Cre,Urine,IntakeVol,Class)
xSet_epi <- model.matrix(~.,xSet_epi)
ySet_epi <- dtNorepi %>%
  dplyr::mutate(Epinephrineflg=as.numeric(Epinephrineflg)) %>%
  pull(Epinephrineflg)
param_list <- list(objective = "binary:logistic",  # For regression
                   eta = 0.02,
                   max_depth = 5,
                   gamma = 0.01,
                   subsample = 0.95
                   )
modEpiRFxgboost <- xgboost::xgboost(data = xSet_epi,
                                   label = ySet_epi,
                        params = param_list, nrounds = 10,
```

```
                        verbose = FALSE,
                        early_stopping_rounds = 8)
# option 2: supply a self-made SHAP values dataset (e.g. sometimes as
output from cross-validation)
SHARP_Epi <- shap.plot.summary(
  data_long = shap.prep(
    xgb_model = modEpiRFxgboost,  X_train = xSet_epi,top_n=25
    ),
  x_bound=0.2)
depSHARP_Epi <- shap.plot.dependence(
  data_long = shap.prep(
    xgb_model = modEpiRFxgboost,  X_train = xSet_epi,top_n=25
    ),
  x = 'Days',
  y = 'Days', color_feature = 'Days') +
  ggtitle("SHAP values of Days vs. Days")
plot_RFxgboost <- ggdraw() +
  draw_plot(SHARP_fluid, x=0,y=0.5, width =  1, height =  0.5) +
  draw_plot(SHARP_Epi, x=0,y=0, width =1, height =  .5)+
  draw_plot_label(c("A", "B"),
                x=c(0,0), y=c(1,.5), size = 15)
```

#External validation with eICU data

```{r eICUvalidation}
LPA_5class_eICU <- dtSepticshock%>%
  dplyr::select(Age,Weight,HR.max,APACHEII,HR.max,BP.min,Temp.max,PH.min,
        HCO3.min,LAC.max,BE.max,PF.min,PCO2.max,Hct,Plt,RDWCV,Cre,Urine)
%>%
  estimate_profiles(n_profiles=5)
dtClass_eICU <- dtSepticshock %>%
  bind_cols(Class=get_data(LPA_5class_eICU)$Class) %>%
  #change class number for ease of comparison: the class membership is
randomly assigned.
  dplyr::mutate(Class = if_else(
    Class==5,3,
    if_else(
      Class ==3,2,
      if_else(
        Class == 4,5,
        if_else(
          Class == 2,4,Class
        )
      )
    )
  ))
laby_eICU <- dtClass_eICU %>%
  mutate(Class=factor(Class,levels = 1:5,
                      labels = paste("Class",1:5))) %>%
  mutate(across(.cols =
c(Age,Weight,HR.max,APACHEII,HR.max,BP.min,Temp.max,PH.min,
        HCO3.min,LAC.max,BE.max,PF.min,PCO2.max,Hct,Plt,RDWCV,Cre,Urine),
              scale)) %>%
```

```
  pivot_longer(cols =
c(Age,Weight,HR.max,APACHEII,HR.max,BP.min,Temp.max,PH.min,
         HCO3.min,LAC.max,BE.max,PF.min,PCO2.max,Hct,Plt,RDWCV,Cre,Urine),
               names_to = "Items",
               values_to = "Scaled value") %>%
  group_by(Items,Class) %>%
  summarise(Mean=mean(`Scaled value`)) %>%
  group_by(Items)%>%
  summarise(Max=max(Mean))
laby_eICU <- laby_eICU[match(dtClass_eICU %>%
                   select(c(Age,Weight,HR.max,APACHEII,HR.max,BP.min,Temp.max,PH.min,
         HCO3.min,LAC.max,BE.max,PF.min,PCO2.max,Hct,Plt,RDWCV,Cre,Urine))
%>%
                   colnames(),
                 laby_eICU$Items),] %>% pull(Max)
plot_Class_eICU <- dtClass_eICU %>%
  mutate(Class=factor(Class,levels = 1:5,
                      labels = paste("Class",1:5))) %>%
  mutate(across(.cols =
c(Age,Weight,HR.max,APACHEII,HR.max,BP.min,Temp.max,PH.min,
         HCO3.min,LAC.max,BE.max,PF.min,PCO2.max,Hct,Plt,RDWCV,Cre,Urine),
                scale)) %>%
  pivot_longer(cols =
c(Age,Weight,HR.max,APACHEII,HR.max,BP.min,Temp.max,PH.min,
         HCO3.min,LAC.max,BE.max,PF.min,PCO2.max,Hct,Plt,RDWCV,Cre,Urine),
               names_to = "Items",
               values_to = "Scaled value") %>%
  ggline(x = "Items", y = "Scaled value", add = "mean_ci",
          color = "Class", palette = "lancet",
         position=position_dodge(width = 0.2),
         error.plot="errorbar")+
  stat_compare_means(aes(group = Class), label = "p.signif",
                     label.y = laby_eICU)+
  theme_pubr(x.text.angle=45)+
  labs(x="")
#prediction in the eICU database for the DTR model
dtDTR_eICU <- dtClass_eICU %>%
  dplyr::select(HR.max,BP.min,Temp.max,Class,Urine,Hct,PF.min,
                PH.min,LAC.max,Age,Gender,APACHEII,IntakeVol,OutputVol,Urine,NorepiEq,
                Cre,RDWCV,days,mort,patientunitstayid)
#prediction for optimal fluid infusion
dtoptFluid_eICU <- NULL
for (iii in 1:5) {
  ii=c(0,1,2,3,7)[iii]
  bb=dtDTR_eICU %>%
    mutate(intercept=1) %>%
    dplyr::filter(days==ii) %>%
    dplyr::select(intercept,HR.max,BP.min, Temp.max, Class,Urine, Hct,
   PF.min, PH.min, LAC.max) %>% as.matrix()%*% out1$psi[[iii]][1:10]
  aa=dtDTR_eICU %>%
    mutate(intercept=1) %>%
    dplyr::filter(days==ii) %>%
    dplyr::select(intercept,HR.max,BP.min, Temp.max, Class,Urine, Hct,
```

```
   PF.min, PH.min, LAC.max) %>% as.matrix()%*% out1$psi[[iii]][11:20]
  dtoptFluid_eICU <- dtoptFluid_eICU %>%
    bind_rows(
      dtDTR_eICU %>% dplyr::filter(days==ii) %>%
      bind_cols(
        optFluid= if_else(exp(-bb/(2*aa))>5000,5000,
                      if_else(exp(-bb/(2*aa))<2000,2000,exp(-bb/(2*aa))))
      )
    )
}

for (iii in c(0,1,2,3,7)) {
  dt11 <-  dtoptFluid_eICU %>%
    dplyr::filter(days==iii)
  modeICU <- glm(hospitaldischargestatus~deltaFluid+I(deltaFluid^2),
              dt11,
           family = binomial)
assign(paste("plot_MortDelta_eICU",iii,sep = "_"),
       visreg::visreg(modeICU,
                      xvar ="deltaFluid",
                      scale = "response",gg=T)+
  labs(x="Difference between Actual and Optimal fluid Intake (ml)",
       y="Probability of Hospital Death",
       title = paste("Day",iii,"(n =",
                      nrow(dt11),")",sep = " "))
)
}
#validation for vasopressor
dtoptEpi_eICU <- NULL
for (iii in 1:5) {
  ii=c(0,1,2,3,7)[iii]
  bb=dtDTR_eICU %>%
    mutate(intercept=1) %>%
    dplyr::filter(days==ii) %>%
    dplyr::select(intercept,HR.max,BP.min, Temp.max, Class,Urine, Hct,
   PF.min, PH.min, LAC.max) %>% as.matrix()%*% DTRmodVaso$psi[[iii]][1:10]
  aa=dtDTR_eICU %>%
    mutate(intercept=1) %>%
    dplyr::filter(days==ii) %>%
    dplyr::select(intercept,HR.max,BP.min, Temp.max, Class,Urine, Hct,
   PF.min, PH.min, LAC.max) %>% as.matrix()%*% DTRmodVaso$psi[[iii]][11:20]
  dtoptEpi_eICU <- dtoptEpi_eICU %>%
    bind_rows(
      dtDTR_eICU %>% dplyr::filter(days==ii) %>%
      bind_cols(
        optEpi= if_else(-bb/(2*aa)>1,1,
                      if_else(-bb/(2*aa)<0,0,-bb/(2*aa)))
      )
    )
}

modeICU <- glm(hospitaldischargestatus~deltaEpi+I(deltaEpi^2),
             dtoptEpi_eICU,
```

```
           family = binomial)
plot_Epi_eICU <-
       visreg::visreg(modeICU,
                      xvar ="deltaEpi",
                      scale = "response",gg=T)+
  labs(x="Difference between Actual and Optimal Norepinephrine (mcg/kg/
min)",
       y="Probability of Hospital Death"
       )
plot_eICUvalidation <- ggdraw() +
  draw_plot(plot_Class_eICU, x=0,y=.7, width =  1, height =  .3) +
  draw_plot(plot_MortDelta_eICU_0, x=0,y=0.35, width =0.333, height =  .35)
+
  draw_plot(plot_MortDelta_eICU_1, x=0.333,y=0.35, width =0.333, height =
.35)+
  draw_plot(plot_MortDelta_eICU_2, x=0.666,y=0.35, width =0.333, height =
.35)+
  draw_plot(plot_MortDelta_eICU_3, x=0,y=0, width =0.333, height =  .35)+
  draw_plot(plot_MortDelta_eICU_7, x=0.333,y=0, width =0.333, height =  .
35)+
  draw_plot(plot_Epi_eICU, x=0.666,y=0, width =0.333, height =  .35)+
  draw_plot_label(c("A", "B","C","D","E","F","G"),
                  x=c(0,0, 0.333,0.666,0,.333,.666),
                  y=c(1,.7, 0.7,0.7,0.35,.35,.35), size = 15)
```
